# Pharmacist-Led Discharge Care to Reduce Postdischarge Health Care Utilization: A Randomized Clinical Trial

**DOI:** 10.1001/jamanetworkopen.2026.0719

**Published:** 2026-03-17

**Authors:** Joshua M. Pevnick, Korey Kennelty, An T. Nguyen, Kallie Amer, Carl T. Berdahl, Galen Cook-Wiens, John Fanikos, Julie Fiskio, Hiroshi Gotanda, James Guan, Andrew J. Henreid, Michelle S. Keller, Eunji M. Ko, Donna W. Leang, Yervant Malkhasian, Lina Matta, Dylan Moriarty, Logan Murry, Annie Muske, Teryl K. Nuckols, Onyeche Oche, Audrienne S. Ortiz, Emily Phung, Nabeel Qureshi, Rita Shane, Shirley Wu, Jeffrey L. Schnipper

**Affiliations:** 1 Cedars-Sinai Medical Center, Los Angeles, California; 2 University of Iowa School of Pharmacy, Iowa City; 3 Vasculearn Network, Brookline, Massachusetts; 4 Hospital Medicine Unit, Division of General Internal Medicine and Primary Care, Brigham and Women’s Hospital, Boston, Massachusetts; 5 Leonard Davis School of Gerontology, University of Southern California, Los Angeles; 6 Department of Medicine, Harvard Medical School, Boston, Massachusetts

## Abstract

**Importance:**

Pharmacist-led peridischarge transitions of care (TOC) interventions reduce adverse drug events after hospitalization. However, health care organizations do not usually see a financial incentive to fund these interventions.

**Objective:**

To test whether pharmacist-led TOC interventions could drive reductions in health care resource utilization after hospital discharge.

**Design, Setting, and Participants:**

This pragmatic randomized clinical trial was conducted in 2 urban teaching hospitals in the US. Participants were hospitalized adults aged 55 years or older taking 10 or more long-term prescribed medications or 3 or more high-risk medications (defined as anticoagulants, antiplatelet agents, or antihyperglycemics including insulin), enrolled between December 23, 2019, and December 30, 2022. Data were analyzed from January 2023 to June 2025.

**Intervention:**

Pharmacist-led peridischarge and postdischarge medication management with patients and their care partners, including medication review, discharge medication reconciliation, and addressing medication adherence and safety. Usual care consisted of obtaining a best possible medication history and conducting an admission medication order reconciliation.

**Main Outcomes and Measures:**

The primary outcome was the proportion of patients with all-hospital unplanned 30-day postdischarge hospital or emergency department (ED) utilization. A sample size of 9776 patients would detect absolute differences of 2.5% from an expected baseline of 27.5%. Secondary end points included same-hospital unplanned utilization and several prespecified subgroup analyses to evaluate effect modification.

**Results:**

A total of 6478 patient hospitalizations were randomized and 6428 (3215 usual care and 3213 intervention) were analyzed; 3265 (50.8%) were among male patients. Patients had a mean (SD) age of 75.5 (10.2) years and were taking a median of 16 (IQR, 12-22) long-term prescription medications and 2 (IQR, 1-3) high-risk medications. Three-quarters of patients (4824 [75.0%]) were discharged home. The per-protocol analysis included 6238 patient encounters, 4472 (71.7%) of which were among patients using fee-for-service Medicare for whom all-hospital utilization claims were obtainable; in this group, no significant reduction was found in the proportion with unplanned 30-day all-hospital utilization (593 of 2242 usual care [26.4%] vs 570 of 2230 intervention [25.6%]; difference, 0.9 percentage points [pp]; 95% CI, −1.7 to 3.5 pp). Among all patients randomized, there was also no significant reduction in same-hospital unplanned 30-day utilization (606 of 3112 usual care [19.5%] vs 579 of 3126 intervention [18.5%]; difference, 1.0 pp; 95% CI, −1.0 to 3.0 pp). Among the 589 patients with low medication adherence and literacy, there was a 10.4 pp (95% CI, 3.4-17.4 pp) absolute reduction in same-hospital unplanned utilization (69 of 240 usual care [28.8%] vs 64 of 349 intervention [18.3%]; *P* = .003) (*P* = .01 for effect modification).

**Conclusions and Relevance:**

Among older adults with polypharmacy, no reduction overall in 30-day unplanned hospital and ED utilization from a pharmacist-led TOC intervention was detected, but a reduction was found among patients with low medication adherence and literacy, suggesting benefit for this subgroup.

**Trial Registration:**

ClinicalTrials.gov Identifier: NCT04071951

## Key Points

**Question:**

Pharmacist-led peridischarge transitions of care (TOC) interventions reduce adverse drug events after hospital discharge, but do they reduce readmissions and other health care utilization?

**Findings:**

This randomized clinical trial included 6478 patient hospitalizations among adults aged 55 years or older with polypharmacy or taking high-risk medications, randomized to either usual care (admission medication reconciliation) or pharmacist-led medication review, discharge medication reconciliation, and addressing of medication adherence and safety. No significant between-group difference was detected in all-hospital unplanned 30-day postdischarge health care utilization.

**Meaning:**

Among older adults with polypharmacy, a pharmacist-led TOC intervention did not reduce 30-day unplanned health care utilization after hospital discharge.

## Teaser

This randomized clinical trial examines whether pharmacist-led transitions-of-care interventions after hospital discharge reduce subsequent readmissions and other health care utilization among patients aged 55 years or older with polypharmacy.

## Introduction

The rate of posthospitalization adverse drug events (ADEs) among older adults has been estimated at 4% to 37%,^1,2^ with recent rates of 5% to 6% found in 2 large medication management clinical trials.^3,4^ Posthospitalization ADEs can result in serious morbidity or death. Furthermore, they are a frequent cause of readmission in older adults.^5,6^ Older adults disproportionately experience posthospitalization ADEs due to predisposing comorbidities such as kidney and cognitive impairment and burdensome medication regimens in terms of polypharmacy, dosing complexity, and ADE risk.^7,8,9^ These issues are compounded in the immediate postdischarge period by increased morbidity and care discontinuity.

Prior research shows that up to one-quarter of postdischarge ADEs are preventable.^10,11^ Furthermore, several teams demonstrated that pharmacy-centric transitions-of-care (TOC) interventions had substantial potential to reduce postdischarge ADEs.^3,4,12,13,14,15,16^ Nonetheless, pharmacist-led TOC interventions remain scarcely used. Lack of use may be attributable to the size of prior studies, which necessitated focus on process improvements rather than clinical and financial end points such as health care utilization.

To convince hospital and health system leaders to fund pharmacist-led TOC interventions, we sought evidence that these interventions would impact health care utilization. Thus, we conducted a pragmatic trial with sufficient power to detect an effect of pharmacist-led TOC interventions on health care utilization after hospital discharge.^17^

## Methods

### Trial Design

We conducted a 2-site randomized clinical trial (RCT) of pharmacist-led TOC interventions. Patients were randomized 1:1 to the intervention or usual care arm, with stratification by study site. We incorporated several pragmatic components, including an intervention supported exclusively by operational funding, broad inclusion criteria, recruitment from a common clinical setting, and deference to pharmacists in tailoring the intervention to each patient. This study was approved by the Cedars-Sinai institutional review board, which granted a waiver of informed consent because the intervention was determined to be low risk and potentially beneficial. We followed the Consolidated Standards of Reporting Trials (CONSORT) reporting guideline.

### Setting

Both sites were high-volume urban academic medical centers in the US with established pharmacy programs, strong leadership support for the trial, and pharmacists experienced in conducting intervention components. One site had an established TOC program employing several pharmacists.

### Inclusion and Exclusion Criteria

Initial inclusion criteria were (1) admission to a medical ward or service by an internist or family medicine physician; (2) age of 65 years or older; and (3) use of 10 or more long-term prescribed medications and/or 3 or more high risk-medications, defined as anticoagulants, antiplatelet agents, or antihyperglycemics including insulin. These medication classes were chosen because prior research showed them to be responsible for most hospitalizations caused by ADEs among older adults.^18^ On October 28, 2020, the minimum age was lowered to 55 years to increase enrollment. Patients we did not enroll included (1) those who were excluded from Medicare’s Hospital Readmissions Reduction Program (HRRP; eg, expected transfer to another acute care facility) to focus on utilization most important to stakeholders, (2) those not expected to fully benefit from adherence-related intervention components (eg, expected to be discharged to a skilled nursing facility, rehabilitation facility, psychiatric facility, or locked facility, where medications are administered by facility staff), (3) those expected to require a more tailored intervention (eg, receiving hospice care, expected to undergo self-directed discharge, or undomiciled), and/or (4) those whose participation would not be conducive to testing study hypotheses (eg, expected to be discharged to another state, enrolled into the study within the past year, expected to have same-day discharge before the intervention could be administered, or expected to receive pharmacist-led TOC interventions regardless of the trial [eg, expected to be seen by a transplant pharmacist]).

### Screening, Enrollment, and Randomization

Patients were enrolled between December 23, 2019, and December 30, 2022. An automated daily report identified potentially eligible patients. After manual electronic health record (EHR) review to determine whether patients met all criteria, study personnel enrolled and randomized patients within 72 hours of hospital admission.

Randomization used REDCap (Vanderbilt University, Nashville, Tennessee), with a separate random sequence for each site preloaded by a statistician (G.C.-W.). The allocation sequence was masked from all other personnel. Pharmacy team members (pharmacists, pharmacy technicians, and pharmacy students) were informed of patients’ randomization status as necessary to provide the intervention or enhanced usual care.

Race and ethnicity, extracted from hospital EHRs, were included in the analysis to study whether all patients might benefit equally from the PHARM-DC intervention. Race categories were American Indian or Alaska Native, Asian, Black or African American, White, and other (included any categories not listed), and ethnicity categories were Hispanic and non-Hispanic.

### Intervention

Based on 3 systematic overviews of systematic reviews addressing medication adherence, medication reconciliation, and polypharmacy,^9,19,20^ Pharmacist Discharge Care (PHARM-DC) Group authors published an evidence-based toolkit^21^ and collaborated with pharmacy leaders and team members across both sites to develop a set of recommended (core) and optional intervention components. Recommended core components included 3 predischarge components (medication review, patient education, and discharge medication reconciliation) and 3 postdischarge components (communication with the outpatient pharmacy, communication with the primary care physician, and a postdischarge follow-up telephone call to the patient). Noncore (optional) components included addressing medication adherence and access, adverse-effect management, motivational interviewing, additional postdischarge telephone calls, and drug level monitoring (eFigure in Supplement 2). If care partners were involved in medication management, pharmacy staff engaged them. CancelRx (discontinuation notice sent to the dispensing pharmacy) was present at both sites.

Researchers and pharmacists from both sites (J.M.P., K.K., A.T.N., K.A., J.G., A.J.H., M.S.K., E.M.K., D.W.L, Y.M., L. Matta, D.M., L. Murray, A.M., O.O., A.S.O., E.P., S.W., J.L.S.) conducted numerous virtual meetings to standardize, optimize, and maintain pharmacists’ execution of intervention components. Meetings began biweekly in 2019 before transitioning to monthly and then quarterly. Content included maximizing enrollment, intervention component tracking, discussions of intervention quality and fidelity, and tracking trial outcomes. Because operational funding supported the intervention, most pharmacy staff members conducting TOC work cared for patients in both study arms. Prior to the study, these pharmacy staff members were well aware of the different intervention components and had delivered them to some patients.

Pharmacists tailored the intervention to each patient, as previously described.^17^ Time-and-motion analysis of 40 hospitalizations found that study pharmacists spent a median of 67 minutes (IQR, 46-90 minutes) per hospitalization on intervention activities,^22^ estimated to cost $101 per hospitalization.

### Enhanced Usual Care

For all trial patients, pharmacy team members sought to obtain a best possible medication history,^23,24^ to conduct admission medication order reconciliation, and to use a previously published instrument to assess the medication adherence and literacy (MedAL) score.^17,25,26^ Pharmacy team members did not seek to deliver additional intervention components to usual care patients but were instructed to accommodate any such requests. One site also used automated postdischarge follow-up telephone calls to assess various issues, including medication-related issues. In rare cases, this required pharmacist follow-up.

### Trial Outcomes

The primary outcome was the proportion of patients with an unplanned rehospitalization or emergency department (ED) visit at any hospital within 30 days of discharge from the index hospitalization. In alignment with Medicare’s HRRP, utilization occurring after an initial planned readmission or observation stay within the 30-day period was not counted in the trial outcomes. Because we used Medicare claims data to measure outside hospital utilization, primary outcome measurement was limited to patients with fee-for-service Medicare. No distinctions were made between observation stays and hospital admissions.

Secondary outcomes (all within 30 days) included (1) proportion of patients with any ED visits or hospitalization at any hospital (ie, all-cause ED or hospital utilization among patients with fee-for-service Medicare), (2) proportion of patients with unplanned utilization at study hospitals (for this “same hospital” outcome, study clinicians [authors J.M.P., A.T.N., C.T.B., H.G., E.M.K., and J.S. and nonauthor collaborator A.C.] reviewed the EHR to categorize utilization as planned or unplanned), (3) each component of utilization separately, and (4) 30-day postdischarge mortality. Same-hospital utilization included local hospitals in each health system.

All-hospital utilization immediately preceded by an ED visit was assumed to be unplanned. Using a subset of 321 same-hospital postdischarge stays that required clinician EHR review for our data and safety monitoring board, we found this definition (presence of an ED visit immediately preceding a hospital stay) to be 100% specific and 89.7% sensitive for classifying postdischarge hospitalizations as unplanned.

We planned a priori comparisons of the following subgroups of patients: those with heart failure, those qualifying for trial inclusion by taking 10 or more medications vs by taking 3 or more high-risk medications, those with low MedAL, those with low socioeconomic status, and those within each study site. We also considered age and sex. To maximize power, the study protocol (Supplement 1) specified the outcome of same-hospital unplanned utilization for all subgroup analyses. Using the same outcome, we also conducted per-protocol analyses for patients who received all core components vs those who did not and for each intervention component separately.

### Data Collection

We obtained patient characteristics and same-hospital utilization data via EHR queries. We assessed intervention fidelity via structured pharmacist documentation. One site leveraged operational documentation workflows, whereas the other site developed new workflows to track fidelity.

All-hospital utilization and mortality were assessed with Medicare claims and National Death Index data accessed via the National Institute on Aging’s Data LINKAGE Program. Our initial intent was to use statewide all-payer claims databases, allowing inclusion of all patients into the primary outcome analysis. Regulatory and other barriers made this plan impracticable.

### Statistical Analysis

We analyzed the primary outcome using a 2-sided *z* test for proportions. Effect modification was evaluated using interaction terms in a multivariable logistic model of same-hospital unplanned utilization by intervention, subgroup, and intervention × subgroup. For these secondary analyses, we used general linear models to cluster by patient for those enrolled more than once and adjusted for common covariates specified a priori, including age, sex, race and ethnicity, median household income by US census tract, Elixhauser Comorbidity Index, MedAL score, and medication eligibility criteria. To account for multiple hypothesis testing, we used the Benjamini-Hochberg procedure. To account for data missing at random, we used multiple imputation in secondary analyses. All analyses were conducted from January 2023 to June 2025, using SAS, version 9.4 (SAS Institute Inc). Using a 2-sided *z* test, 80% power, and α of .05, our targeted sample size of 9776 patients would detect absolute differences of 2.5% from an expected baseline primary outcome of 27.5%. Two-sided *P* < .05 was considered significant.

## Results

### Enrollment, Randomization, and Baseline Characteristics

**Figure 1.**
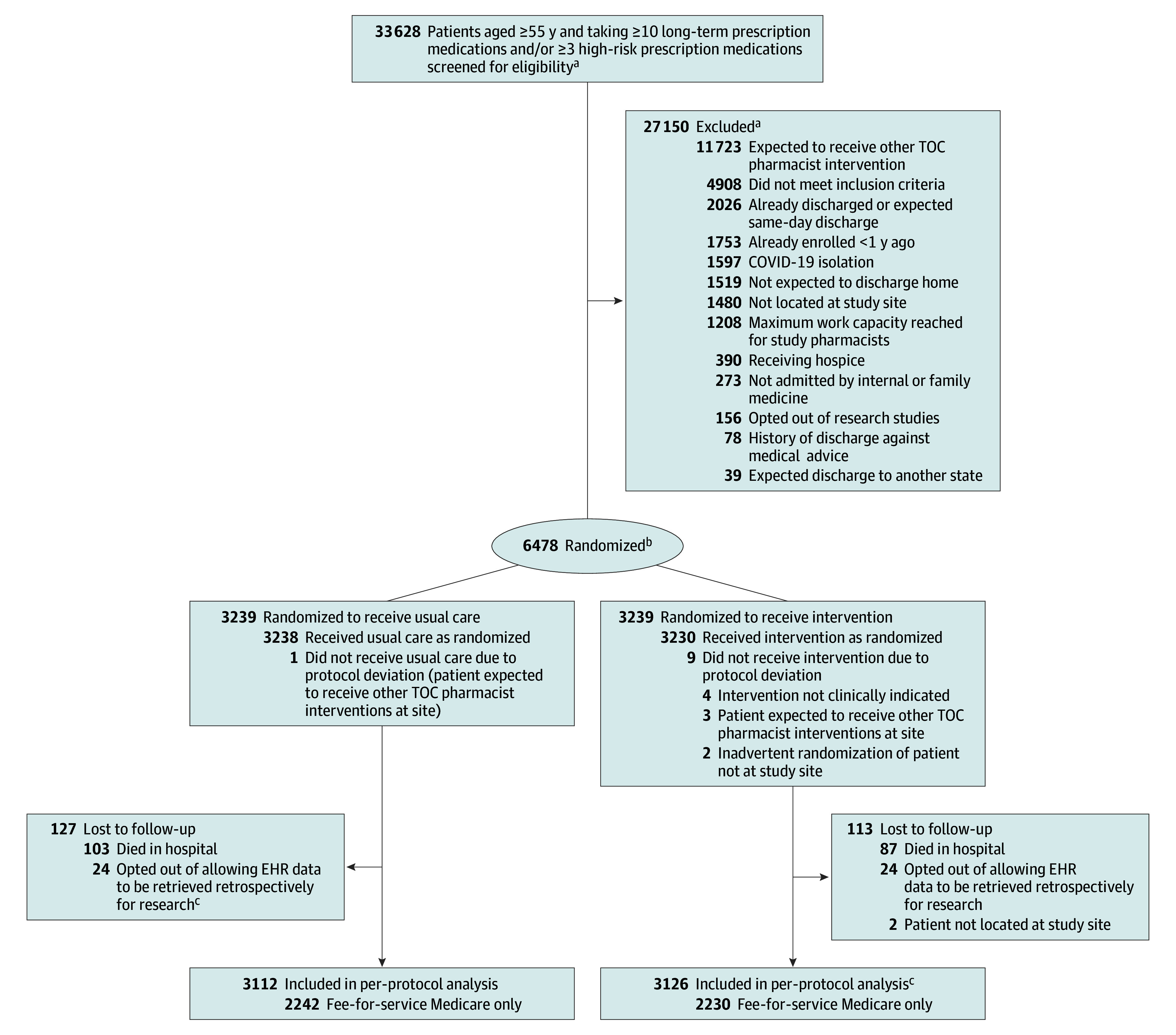
Study Flow Diagram EHR indicates electronic health record; TOC, transition of care. ^a^To estimate the number of patients screened for eligibility, we calculated the product of the total number of workdays during the study period and the mean number of patients screened per workday. The latter was estimated using a random audit of study documentation on 2 days per quarter across the study period. Additionally, this audit was used to estimate the proportion of patients excluded for each reason listed. ^b^Nine patients were inadvertently randomized twice into the study who had already been randomized within 1 year prior (an exclusion criterion). They were included in the analysis. ^c^Patients who had opted out of participating in any research at the time of enrollment were not enrolled. However, after the trial ended, as part of the process of extracting outcomes, we realized that some enrolled patients had opted out of research after they had been enrolled. Because outcomes could not be obtained, they were excluded from analyses.

We screened 33 628 patients and enrolled 6264 across 6478 randomized hospitalizations (207 patients were randomized twice and 7 were randomized thrice, representing 3.2% and 0.1% of encounters, respectively) (Figure 1). Exclusions most commonly stemmed from expected provision of other TOC pharmacist interventions (largely for operational initiatives), not meeting inclusion criteria, occurrence or expectation of prompt discharge, and previous enrollment in the prior year. Enrollment was limited by the COVID-19 pandemic, availability of pharmacists to conduct the intervention, and availability of eligible patients. In the context of underenrollment, expiring funding, concerns about how long it would take to achieve the intended sample size, recalculation of power and detectable effect size, and suspicion that there was and would continue to be no significant effect for the primary outcome and with input from the data safety monitoring board, trial enrollment was ceased on December 30, 2022. For assessment of the primary outcome, Medicare claims data were available for 2242 of 3112 patient encounters (72.0%) in the usual care arm and 2230 of 3126 (71.3%) in the intervention arm.

Arms were well balanced in nearly all measured baseline characteristics (Table 1). Of the 6428 patient encounters that were analyzed (3215 usual care and 3213 intervention), site 1 enrolled 3829 (59.6%) and site 2, 2599 (40.4%). Mean (SD) patient age was 75.5 (10.2) years; 3163 (49.2%) were female and 3265 (50.8%) were male. Fourteen patients (0.2%) were American Indian or Alaska Native; 257 (4.0%), Asian; 954 (14.8%), Black or African American; 4670 (72.7%), White; 444 (6.9%), other race; and 89 (1.4%), unknown race. A total of 601 patients (9.3%) were Hispanic, 5769 (89.7%) were non-Hispanic, and 58 (0.9%) had unknown ethnicity. EHR data showed that an interpreter was needed for 1233 patients (19.2%). Most patients had fee-for-service Medicare (4602 [71.6%]), followed by Medicare Advantage (820 [12.8%]), commercial insurance (690 [10.7%]), and Medicaid (294 [4.6%]).

**Table 1.** Patient Demographics and Key Baseline Characteristics at Randomization

Characteristic	Patients, No (%) (N = 6428)	
	Usual care (n = 3215)	Intervention (n = 3213)
Study site		
1	1915 (59.6)	1914 (59.6)
2	1300 (40.4)	1299 (40.4)
Age, y		
Mean (SD)	75.6 (10.2)	75.5 (10.2)
55-64	466 (14.5)	472 (14.7)
65-74	1039 (32.3)	1055 (32.8)
75-84	1033 (32.1)	1006 (31.3)
≥85	677 (21.1)	680 (21.2)
Sex		
Female	1577 (49.1)	1586 (49.4)
Male	1638 (50.9)	1627 (50.6)
Race		
American Indian or Alaska Native	8 (0.2)	6 (0.2)
Asian	118 (3.7)	139 (4.3)
Black or African American	475 (14.8)	479 (14.9)
White	2367 (73.6)	2303 (71.7)
Other^a^	201 (6.3)	243 (7.6)
Unknown	46 (1.4)	43 (1.3)
Ethnicity		
Hispanic	308 (9.6)	293 (9.1)
Non-Hispanic	2870 (89.3)	2899 (90.2)
Unknown	37 (1.2)	21 (0.7)
Interpreter needed		
No	2581 (80.3)	2522 (78.5)
Yes	590 (18.4)	643 (20.0)
Unknown	44 (1.4)	48 (1.5)
Insurance at index admission		
Commercial	342 (10.6)	348 (10.8)
Fee-for-service Medicare	2314 (72.0)	2288 (71.2)
Medicaid	132 (4.1)	162 (5.0)
Medicare Advantage	411 (12.8)	409 (12.7)
Self-pay or other insurance	16 (0.5)	6 (0.2)
Household income by US census tract		
Data available	3101 (96.5)	3059 (95.2)
Median (IQR), $	92 353 (65 592-119 159)	93 360 (68 020-120 879)
Same-hospital stays during the 3 y prior to randomization, median (IQR), No.	1 (0-3)	1 (0-3)
Length of stay, median (IQR), d	5.8 (3.3-9.9)	5.8 (3.3-9.9)
Discharge destination		
Home	2385 (74.2)	2439 (75.9)
Skilled nursing facility	332 (10.3)	320 (10.0)
Inpatient rehabilitation hospital	223 (6.9)	224 (7.0)
Died	103 (3.2)	87 (2.7)
Hospice	86 (2.7)	73 (2.3)
Other facility	63 (2.0)	45 (1.4)
Against medical advice	23 (0.7)	25 (0.8)
MedAL		
Low	245 (7.6)	357 (11.1)
Moderate	598 (18.6)	749 (23.3)
High	984 (30.6)	1129 (35.1)
Not documented	872 (27.1)	466 (14.5)
No intent to assess with MedAL^b^	516 (16.0)	512 (15.9)
Prescribed medications prior to hospital admission		
Median (IQR), No.	17 (13-22)	16 (12-22)
≥10 Long-term	2467 (76.7)	2657 (82.7)
≥3 High-risk^c^	901 (28.0)	917 (28.5)
Both ≥10 long-term and ≥3 high-risk	856 (26.6)	854 (26.6)
Missing^d^	497 (15.5)	154 (4.8)
Elixhauser Index, mean (SD)	13.8 (10.0)	13.9 (10.2)
Comorbidities		
Heart failure	846 (26.3)	820 (25.5)
Dementia	129 (4.0)	113 (3.5)
Metastatic cancer	69 (2.1)	91 (2.8)
Severe kidney disease	200 (6.2)	174 (5.4)
Diabetes	695 (21.6)	641 (20.0)
Coronary artery disease	802 (24.9)	750 (23.3)

Abbreviation: MedAL, medication adherence and literacy.

^a^ Other included any categories other than those listed.

^b^ Enrolled after MedAL assessment stopped being used at 1 enrolling site.

^c^ Anticoagulants, antihyperglycemics, and antiplatelet agents.

^d^ In cases with missing pharmacy technician admission medication history notes, study inclusion was based on polypharmacy documented in recent outpatient notes. Because the number of medications was not recorded at enrollment but rather extracted from pharmacy technician admission medication history notes, it could not be included here. Higher missingness among usual care patients may have stemmed from less interaction with pharmacist team members.

Patients were taking a median of 16 (IQR, 12-22) long-term prescribed medications and 2 (IQR, 1-3) high-risk medications. Three-quarters of patients (4824 [75.0%]) were discharged home. Despite the intention to enroll only patients expected to be discharged home, 652 (10.1%) were discharged to a skilled nursing facility and 447 (7.0%) to an acute rehabilitation hospital.

Data missingness was infrequent and generally balanced across arms. One notable exception involved MedAL scores. This occurred for multiple reasons. First, 1 site changed its assessment method starting on January 5, 2022. This was driven by competing operational obligations and concerns about interrater reliability of medication literacy assessments performed by pharmacy technicians. Even after excluding patients affected by these changes, MedAL scores were missing for 872 patients (27.1%) in the usual care arm vs 466 (14.5%) in the intervention arm. Missingness rates were nearly identical on the day of admission. Thus, the increase in MedAL documentation for patients in the intervention arm occurred later during hospitalization, likely due to increased interaction between pharmacy team members and intervention patients.

### Intervention Fidelity and Contamination

**Figure 2.**
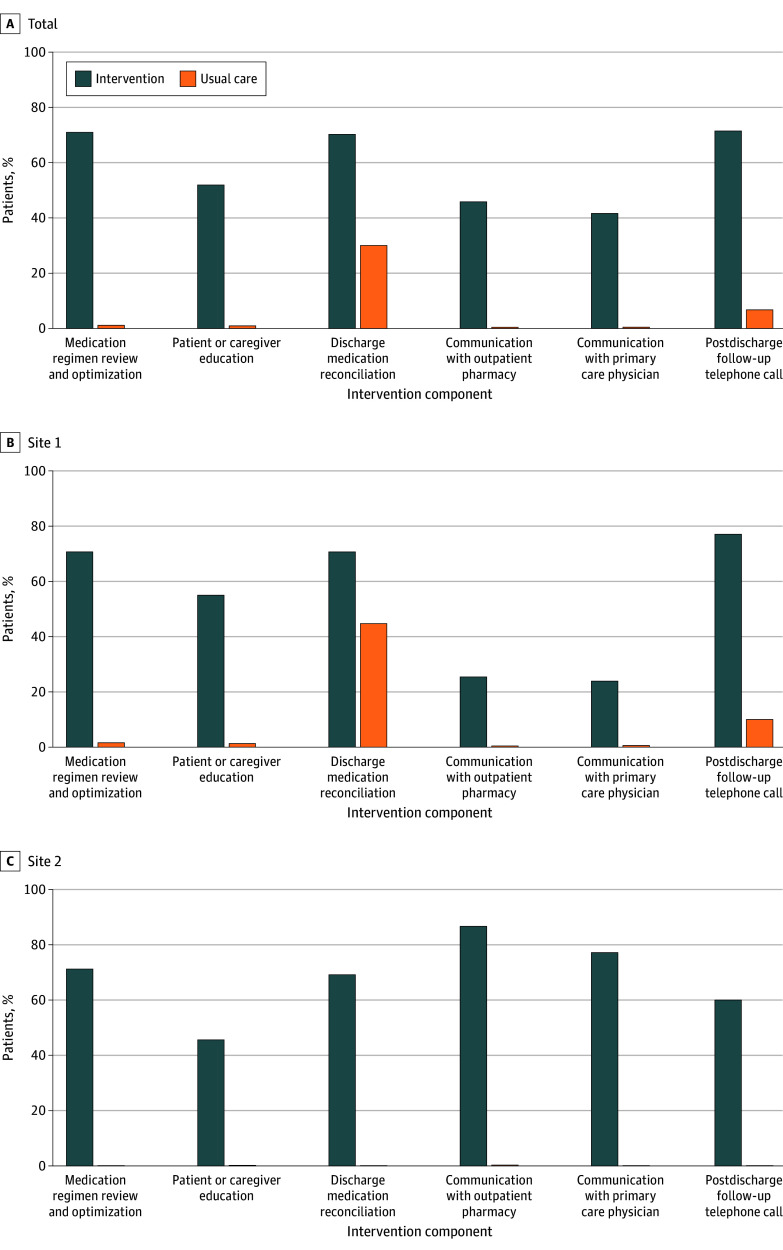
Bar Graph Showing Receipt of Core Components by Study Arm Data on core component receipt were available for all 3829 patients enrolled at site 1, whereas at site 2, technical constraints prevented collection of core component receipt data until September 2020. Thus, panel A reflects a denominator of only 5796 patients (2899 in the intervention and 2897 in the control arm), representing 90.2% of all 6428 patient encounters analyzed.

Among 2899 intervention patients with complete data, pharmacists documented receipt of intervention core components ranging from 1225 patients (42.3%) to 2103 (72.5%) (Figure 2A). Implementation was highest for postdischarge follow-up telephone calls and lowest for communication with primary care physicians and outpatient pharmacies. Figure 2B shows that contamination (ie, receipt of intervention components among usual care patients) occurred predominantly due to operational initiatives at 1 site and among 2 intervention components: discharge medication reconciliation (882 of 2897 patients [30.4%]) and postdischarge follow-up telephone calls (198 of 2897 patients [6.8%], with efforts directed toward patients with low MedAL). Admission medication reconciliation, which was intended for all patients, was received by 2293 of 2899 patients (79.1%) in the intervention arm. We estimated 73.1% receipt in the usual care arm, though this was carefully tracked at only 1 site.

### Primary and Related Outcomes

Out of 6238 patient encounters in the per-protocol analysis, 4472 (71.7%) were among patients with fee-for-service Medicare for whom all hospital utilization claims were available; of those, unplanned all-hospital utilization occurred in 593 of 2242 (26.4%) in the usual care arm vs 570 of 2230 (25.6%) in the intervention arm. The absolute difference was 0.9 percentage points (pp) (95% CI, −1.7 to 3.5 pp; risk ratio, 0.97; 95% CI, 0.88-1.07) (Table 2).

**Table 2.** Primary Outcome and Related Results

Outcome	Patients, No./total No. (%)		Absolute reduction, pp (95% CI)	*P* value
	Usual care	Intervention		
Primary outcome: all-hospital 30-d unplanned ED or hospital utilization (n = 4472)^a^^,^^b^	593/2242 (26.4)	570/2230 (25.6)	0.9 (−1.7 to 3.5)	.50
All-hospital all-cause 30-d ED or hospital utilization (n = 4472)^b^	610/2242 (27.2)	595/2230 (26.7)	0.5 (−2.1 to 3.1)	.69
Same-hospital 30-d unplanned ED or hospital utilization (n = 6238)^c^	606/3112 (19.5)	579/3126 (18.5)	1.0 (−1.0 to 3.0)	.34

Abbreviations: ED, emergency department; pp, percentage points.

^a^ Unplanned utilization at outside hospitals was determined by excluding hospital stays not preceded by an ED visit.

^b^ A total of 1766 patients (870 usual care, 896 intervention) were excluded due to not having fee-for-service Medicare that allowed assessment of utilization at non–study site hospitals via Centers for Medicare & Medicaid Services claims.

^c^ Unplanned utilization was assessed via electronic health record review.

### Secondary Outcomes

There were no statistically significant differences in all-hospital, all-cause (planned or unplanned) 30-day utilization. There was also no significant difference in same-hospital unplanned 30-day utilization events (606 of 3112 usual care [19.5%] vs 579 of 3126 intervention [18.5%]; difference, 1.0 pp; 95% CI, −1.0 to 3.0 pp) (Table 2). Similarly, eTable 1 in Supplement 2 shows no significant differences in utilization outcomes evaluated separately or in 30-day postdischarge mortality. Although the *P* value for same-hospital 30-day unplanned ED utilization was .03 (between-group difference, 1.3 pp; 95% CI, 0.2-2.5 pp), this exceeded the Benjamini-Hochberg statistical significance threshold of .014. No per-protocol comparisons were significant.

### Subgroup Analyses

**Figure 3.**
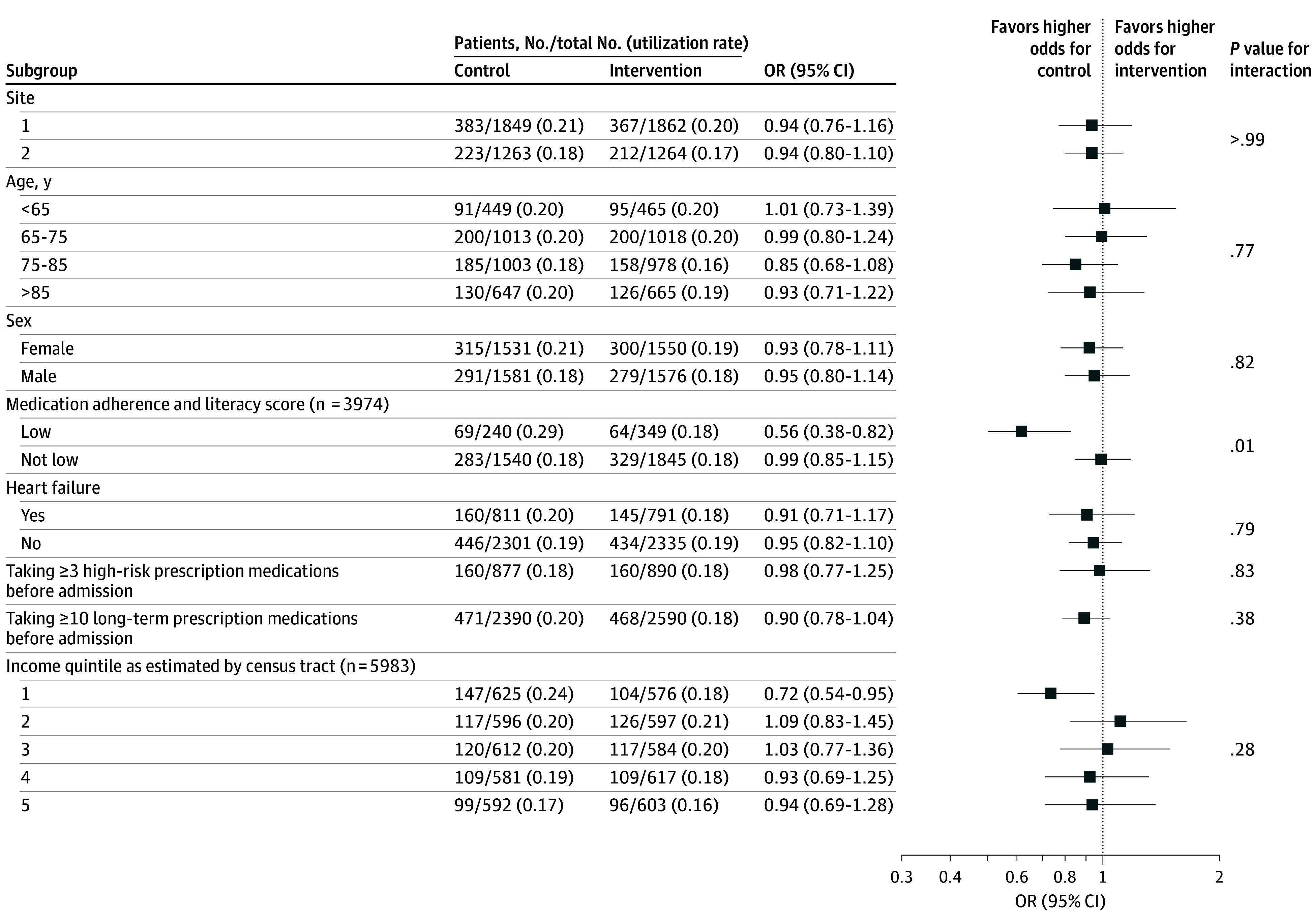
Forest Plot of Intervention Effect on Odds of Unplanned Same-Hospital Utilization (n = 6238) by Subgroup OR indicates odds ratio.

Figure 3 shows the effect of the intervention on a priori–specified subgroups. eTable 2 in Supplement 2 shows that the intervention caused a 10.4 pp (95% CI, 3.4-17.4 pp; *P* = .003) absolute reduction in same-hospital unplanned utilization among the 589 patients with low MedAL (69 of 240 [28.8%] usual care vs 64 of 349 [18.3%] intervention; odds ratio [OR], 0.56 [95% CI, 0.38-0.82]). We found evidence of effect modification by low MedAL (*P* = .01 for the interaction term of arm × low vs not low MedAL). The Benjamini-Hochberg procedure confirmed statistical significance, even accounting for multiple hypothesis testing (eTable 3 in Supplement 2).^27^ The effect was larger (absolute reduction, 13.0 pp; 95% CI, 3.2-22.8 pp) at the site with less contamination than at the site with more contamination (8.2 pp; 95% CI, −1.7 to 18.2 pp). Although there was some evidence of benefit among patients in the lowest quintile of median income, this effect was less robust than for the MedAL score and exceeded the Benjamini-Hochberg threshold for statistical significance. We found no other evidence of effect modification, including in post hoc analyses of patients discharged to home and during time periods associated with COVID-19 hospitalization surges.

We used multiple imputation to address MedAL missingness that occurred after 1 site stopped using MedAL scores, because these data were missing at random. Using a simple model that only included MedAL categories of low, not low, and otherwise missing (not imputed, because they were not missing at random), we found that patients with low MedAL randomized to the intervention had an OR of 0.60 (95% CI, 0.40-0.88; *P* = .009) for 30-day same-hospital postdischarge utilization as compared with usual care (eTable 4 in Supplement 2). Adjusting for relevant covariates yielded similar estimates (adjusted OR, 0.61; 95% CI, 0.42-0.90; *P* = .01).

## Discussion

In this pragmatic RCT, a pharmacist-led peridischarge TOC intervention was not found to decrease all-hospital unplanned acute care utilization at 2 large academic centers when added to existing robust admission medication reconciliation programs. The single most relevant comparison is a 2023 Cochrane systematic review analyzing medication regimen review.^28^ The 17 trials included in this review were similar to ours in terms of intervention components and target populations. One notable difference was a median follow-up of 6 months in the review. This may have increased sensitivity to detect benefit.

A quantitative meta-analysis of 9561 patients found that trialed interventions “likely reduced” all-cause hospital readmissions (risk ratio, 0.93; 95% CI, 0.89-0.98) with “moderate-certainty evidence.”^28^ The risk ratio of 0.93 was similar to that of our study (0.97; 95% CI, 0.88-1.07).

Even though the point estimate of 0.9 pp from our trial could indicate an operationally significant reduction in utilization, there are 5 reasons why our trial may not have achieved the statistical significance detected in the meta-analysis.^28^ These include underenrollment (further limited by inability to collect all-hospital utilization data on patients without Medicare fee-for-service insurance), such that the sample did not achieve 80% power to detect the prespecified effect size; imperfect fidelity; contamination; known deficiencies in health care utilization as a marker of high-quality transitional care^29^; and receipt of a best possible medication history and admission medication reconciliation in both arms. Prior studies demonstrate the potency of these 2 interventions to improve medication safety.^30,31^ Using Baker and colleagues’^31^ taxonomy of medication reconciliation, the usual care group in the current study would be “silver” level, whereas the intervention group would be “diamond.”

In terms of the best target population, our trial offers, to our knowledge, the strongest evidence to date that patients with low MedAL may benefit most from pharmacist-led postdischarge medication management. This result proved robust to accounting for multiple comparisons. Furthermore, using multiple imputation to account for data missing at random did not substantially alter the effect size or statistical significance. In addition, because operationally motivated postdischarge follow-up telephone calls were directed to patients with low medication adherence in the control group at 1 site, our results may underestimate the true effect.

Given the medication regimen complexity of included patients and given that many intervention components sought to improve communication of medication information, it is intuitive that patients with suboptimal MedAL would benefit most. In other words, if the barrier to postdischarge medication management is lack of knowledge due to low health literacy, that gap may be bridged with effective pharmacist-led education, counseling, and follow-up. At least 1 post hoc analysis of a similar intervention found an association between suboptimal medication literacy and reduced health care utilization.^32^ To our knowledge, ours is the first trial to show benefit in an a priori subgroup. Based on this result, operational work and trials should focus on patients with low MedAL. These patients represented less than 10% of those enrolled in our trial. Thus, targeting this population should lessen costs and increase feasibility. Pharmacist-led, patient-directed interventions may also help hospitals meet accreditation standards for medication reconciliation, an area with which they have historically struggled.^33,34^

### Strengths and Limitations

One strength of this study is its pragmatic nature, such that its estimates of benefit apply to typical clinical settings. We also acknowledge several limitations. First, the 5 aforementioned factors (underenrollment, contamination, imperfect fidelity, known deficiencies in health care utilization as a marker of high-quality transitional care, and intentional provision of important TOC interventions to usual care patients) may have caused type II error. Second, without access to EHR data from outside hospitals, we had to use ED visits as a proxy for unplanned utilization for the primary outcome (but not for the same-hospital utilization outcome). Third, we expanded enrollment criteria over time. Fourth, results may not generalize to hospitals with less robust pharmacy support; limited pharmacist availability to enroll patients and the COVID-19 pandemic may also have affected the study population and, thus, generalizability. Fifth, changing methods of MedAL assessment over time and differential missingness weaken the quality of evidence showing a benefit in the subgroup with low MedAL. Future operational work may benefit from a better way to measure health literacy at hospital admission (eg, the BRIEF Health Literacy Screening Tool).^35^ Sixth, we did not present data on cognitive impairment, dementia, or care partner engagement. Future studies should seek to identify affected patients and their care partners and to tailor intervention components to them. Seventh, the large size of our trial precluded more detailed measurement of fidelity and contamination (eg, via direct observation). We do not believe that having the same pharmacists care for both intervention and control patients led to additional contamination; all these pharmacists had provided these interventions to some patients prior to the study. During the study, they did not have time to deliver additional interventions to control patients unless asked (in which case, it was tracked).

## Conclusions

In this pragmatic RCT, a pharmacist-led peridischarge TOC intervention was not found to decrease all-hospital unplanned acute care utilization after discharge from the index hospitalization among patients aged 55 years or older with polypharmacy at 2 large academic centers when added to robust medication-reconciliation programs existing at admission. Because an a priori subgroup analysis of patients with low MedAL appeared to show benefit, health care organizations seeking to improve patient outcomes and reduce avoidable health care utilization after hospital discharge should consider adopting these interventions for this subgroup. Because patients with low MedAL represented less than 10% of those enrolled at 2 large academic centers, similar organizations may find that targeting this population lessens costs and increases feasibility.

## Notes

Supplement 1.
Trial Protocol

Supplement 2. **eFigure.** Patient Flow **eTable 1.** Utilization Outcomes Evaluated Separately and Postdischarge Mortality **eTable 2.** Same-Hospital 30-Day Unplanned Utilization Stratified by Medication Adherence and Literacy (MedAL) Score **eTable 3.** Tests for Effect Modification **eTable 4.** Effect of Intervention on Postdischarge Utilization Among Patients With Low Medication Adherence and Literacy
eReference

Supplement 3.
Nonauthor Collaborators

Supplement 4.
Data Sharing Statement
