# Supplementary material for: Pharmacist-Led Discharge Care to Reduce Postdischarge Health Care Utilization: A Randomized Clinical Trial
Source: JAMA Netw Open. 2026 Mar 17;9(3):e260719. doi: 10.1001/jamanetworkopen.2026.0719 (PMC13446754; doi:10.1001/jamanetworkopen.2026.0719)
Supplement: Supplementary file 1 [file jamanetwopen-e260719-s001.pdf]

PHARMacist Discharge Care (PHARM-DC)

Study Protocol

PHARMacist Discharge Care (PHARM-DC)

Protocol Number: 3.0

National Clinical Trial (NCT) Identified Number: NCT04071951

Principal Investigator: Dr. Joshua Pevnick

Funded by: National Institute of Aging

Version Number: 16.0

2023.08.25

Table of Contents

STATEMENT OF COMPLIANCE 1
1 PROTOCOL SUMMARY 1
1.1 Synopsis 1
1.2 Specific aims 3
1.3 Schedule of Activities (SoA) 5
2 INTRODUCTION 7
2.1 Study Rationale 7
2.2 Background 8
2.3 Risk/Benefit Assessment 11
2.3.1 Known Potential Risks 11
2.3.2 Known Potential Benefits 12
3 OBJECTIVES AND ENDPOINTS 13
4 STUDY DESIGN 14
4.1 Overall Design 14
5 STUDY POPULATION 15
5.1 Inclusion Criteria 15
5.2 Exclusion Criteria 16
5.3 Strategies for Recruitment and Retention 16
6 STUDY INTERVENTION 17
6.1 Study Intervention(s) Administration 17
6.1.1 Study Intervention Description 17
6.2 Measures to Minimize Bias: Randomization and Blinding 18
6.3 Study Intervention Compliance 18
7 STUDY INTERVENTION DISCONTINUATION AND PARTICIPANT DISCONTINUATION/WITHDRAWAL 19
7.1 Lost to Follow-Up 19
7.2 Adverse Events and Serious Adverse Events 19
7.2.1 Definition of Adverse Events (AE) 19
7.2.2 Definition of Serious Adverse Events (SAE) 19
7.2.3 Classification of an Adverse Event 19
7.2.4 Time Period and Frequency for Event Assessment and Follow-Up 20
8 STATISTICAL CONSIDERATIONS 20
8.1 Statistical Hypotheses 20
8.2 Sample Size Determination 20
8.3 Populations for Analyses 21
8.4 Statistical Analyses 21
8.4.1 General Approach 21
8.4.2 Analysis of the Secondary Endpoint(s) 22
8.4.3 qualitative Analyses 22
8.4.4 Cost-effectiveness analyses 23
8.4.5 Secondary data analysis: Examine effects of PHARM-DC intervention on initiation and deprescribing of benzodiazepine-receptor agonists 25
8.4.6 Secondary data analysis: Examine effects of PHARM-DC intervention on fall-related injuries among older adults with Alzheimer’s disease and related dementias 25
8.4.7 Secondary data analysis: PROPENSITY SCORE ANALYSIS at BRIGHAM AND WOmen’s hospital 26

9 SUPPORTING DOCUMENTATION AND OPERATIONAL CONSIDERATIONS 27
9.1 Regulatory, Ethical, and Study Oversight Considerations 27
9.1.1 Informed Consent Process 27
9.1.2 Confidentiality and Privacy 30
9.1.3 Key Roles and Study Governance 31
9.1.4 Safety Oversight 31
9.1.5 Data Handling and Record Keeping 32
9.1.6 Publication and Data Sharing Policy 32
9.1.7 Conflict of Interest Policy 33
9.2 Protocol Amendment History 34
10 APPENDIX A 37
11 REFERENCES 38

STATEMENT OF COMPLIANCE

(1) [The trial will be carried out in accordance with International Conference on Harmonisation Good Clinical Practice (ICH GCP) and the following:

- United States (US) Code of Federal Regulations (CFR) applicable to clinical studies (45 CFR Part 46, 21 CFR Part 50, 21 CFR Part 56, 21 CFR Part 312, and/or 21 CFR Part 812)

National Institutes of Health (NIH)-funded investigators and clinical trial site staff who are responsible for the conduct, management, or oversight of NIH-funded clinical trials have completed Human Subjects Protection and ICH GCP Training.

The protocol, recruitment materials, and all participant materials will be submitted to the Institutional Review Board (IRB) for review and approval. Approval of both the protocol and information sheets will be obtained before any participant is enrolled. Any amendment to the protocol will require review and approval by the IRB before the changes are implemented to the study. In addition, all changes to the information sheets will be IRB-approved.

1 PROTOCOL SUMMARY

1.1 SYNOPSIS

Title: PHARMacist Discharge Care (PHARM-DC)

Study Description:

To implement evidence-based pharmacist-led strategies for preventing ADEs after hospitalization, use implementation science methodology to study implementation at two large medical centers, and examine the cost-effectiveness of the intervention.

There are three components to this study: (1) the prospective, randomized trial, which includes a pharmacist-led hospital discharge care intervention, (2) the qualitative study, which involves focus groups and interviews, and (3) a cost-effectiveness study, which involves a review of the literature and a time-and-motion study component.

Objectives:

(1) Pharmacist-led Hospital Discharge Care Intervention: To test the effect of **PHARMacist Discharge Care** (**PHARM-DC**) Toolkit on post-discharge utilization among patients most at risk for post-discharge adverse drug events (ADEs): recently discharged older adults taking ≥10 chronic prescription medications or ≥3 high-risk medications.

(2) Qualitative study: To study barriers and facilitators of implementing PHARM-DC at two sites, Cedars-Sinai Medical Center (CSMC) and Brigham and Women's Hospital (BWH) using a qualitative study.

(3) Cost-Effectiveness Study: To analyze the costs of PHARM-

DC, including the incremental cost per readmission averted and the net incremental cost from the health system perspective using a time-and-motion study and cost-effectiveness analysis.

Endpoints:

Pharmacist-led Hospital Discharge Care Intervention:
Primary Endpoint: 30-day post-discharge utilization, including same-hospital utilization data and same-state 30-day hospital readmissions and ED visits.

Secondary Endpoints: To assess the *Reach* of the intervention, we will examine differences in effectiveness across sites, and for patients of different ages, sex, taking different numbers of medications, with different comorbidities/diagnoses, and living in zip codes reflecting different median incomes.

Study Population:

The study population includes individuals being discharged from the hospital at high risk for adverse drug events. Inclusion criteria includes individuals being discharged from a medical/surgical ward and either being 55 years of age or older and having 10 or more chronic prescription medications, **or** using three or more high-risk medications before or during hospitalization.

Description of Sites/Facilities Enrolling Participants:

Two sites will participate in this study: an academic medical center (Brigham and Women's Hospital in Boston, Massachusetts) and a university-affiliated community hospital (Cedars-Sinai Medical Center in Los Angeles, California [1]) that has some community physicians.

Description of Study Intervention:

Pharmacist-led Hospital Discharge Care Intervention:

Eligible pharmacists (those with training in transitions of care) at the two sites will receive a list of patients currently admitted to the hospital who meet the inclusion/exclusion criteria. Eligible patients will meet with a clinical pharmacist for medication reconciliation.

Clinical pharmacists will conduct an initial medication regimen assessment, which will use tools in the PHARM-DC Toolkit to identify potentially inappropriate medications. Patients and/or their caregivers will undergo a pre-discharge intervention, where clinical pharmacists will anticipated discharge medication changes.

The Toolkit content and 1-2 hours of introductory training add three major components:

1. Pharmacists will use the ***PHARM-DC Toolkit,*** which ***summarizes the evidence*** for post-discharge medication management. The Toolkit includes a (1) the Start Actions for

Assessing Adherence Barriers worksheet, integrated into an Epic template, (2) the Beers Medication List, (3) Pre-Discharge Worksheet, integrated into an Epic template, (4) Up-to-Date Section on prescribing to older adults, (5) Discharge Worksheet, integrated into an Epic template, (6) Post-Discharge Worksheet, integrated into an Epic template.

2. Pharmacists will *assess patient needs* and *customize accordingly*. All patients will receive one discharge counseling visit and one post-discharge phone call, but pharmacists will find that some patients need further phone calls, interventions, referrals, or other interactions.

   During training, trainers will emphasize to pharmacists participating in this study additional, clinically-indicated services may be provided to control group patients to ensure that it is clear that routine services that are part of the PHARM-DC and that the toolkit should not be withheld from the control group if they are clinically indicated.

Study Duration: 36 months
Participant Duration: Participation during one hospital admission/discharge

1.2 SPECIFIC AIMS

Our PHARM-DC toolkit improves upon the intervention in our prior trial by incorporating evidence-based pharmacist-led strategies for preventing ADEs after hospitalization. To go beyond just instructing pharmacists to counsel and call patients, we systematically searched the literature for medication management interventions addressing medical conditions most likely to lead to readmission among older adults taking ≥10 chronic prescription medications or ≥3 high-risk medications. As such, the toolkit incorporates new evidence involving deprescribing interventions to address polypharmacy, as well as from a multidisciplinary technical expert panel.

This study uses a pragmatic trial randomized at the patient level and conducted in two large hospitals to achieve the following aims, each of which has been designed using the RE-AIM framework:

Aim 1: To test the effect of PHARM-DC on post-discharge utilization among patients most at risk for post-discharge ADEs: recently discharged older adults taking ≥10 medications or ≥3 high-risk medications using a prospective, randomized, pragmatic multi-site study.

Aim 2: To study barriers and facilitators of implementing PHARM-DC using a qualitative study.

Aim 3: To analyze the costs of PHARM-DC, including the incremental cost per readmission averted and the net incremental cost from the health system perspective using a time-and-motion study and a cost-effectiveness analysis.

Figure 1: Flow diagram for PHARM-DC Discharge Intervention

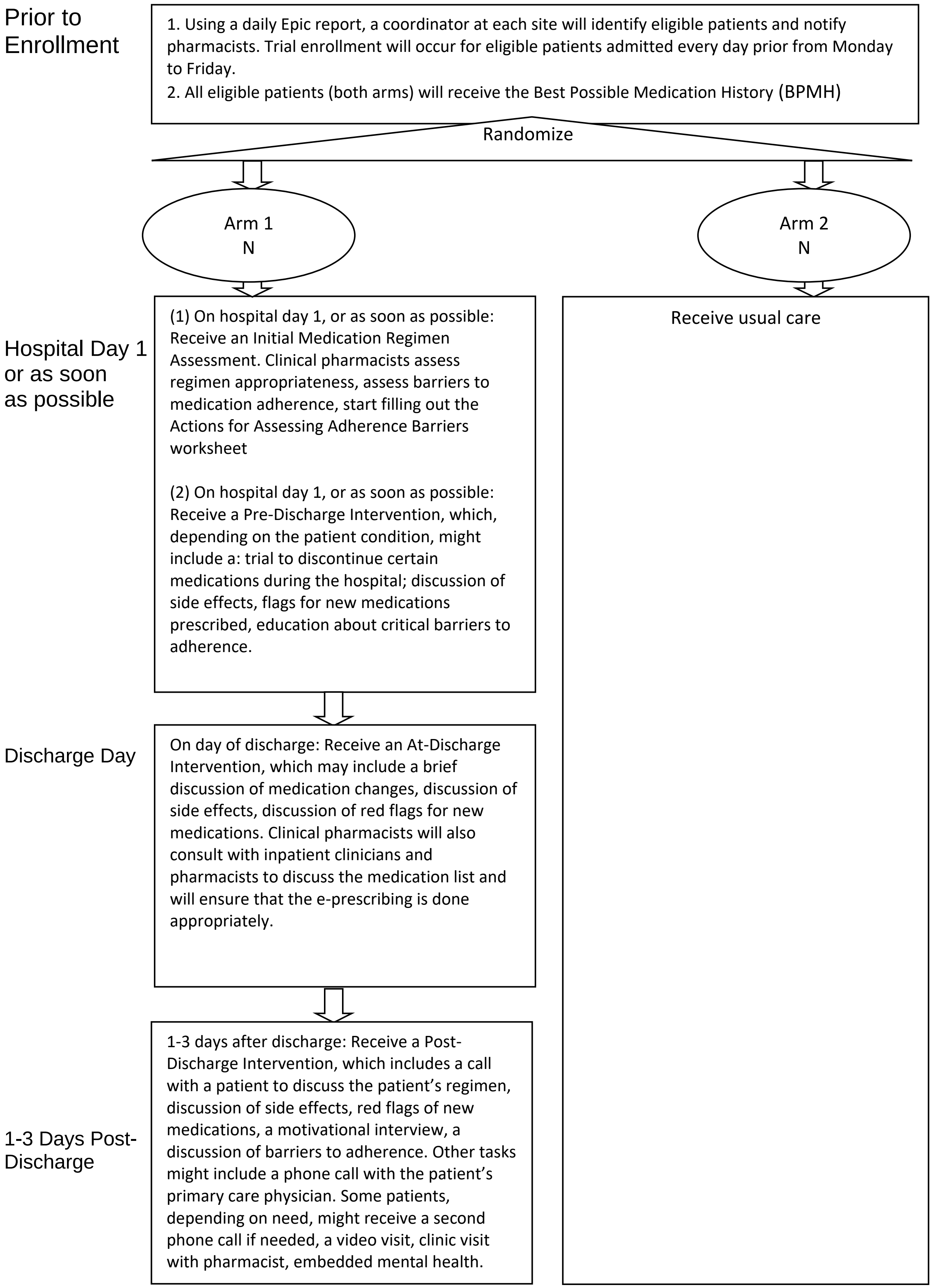

1.3 SCHEDULE OF ACTIVITIES (SOA)

(1) Pharmacist-led Hospital Discharge Care Intervention:

a. Eligible pharmacists at each study site will be selected to participate in the intervention based on their training in transitions of care.
b. Using a daily Epic report generated by EIS, a coordinator at each site will identify eligible patients and notify pharmacists. Trial enrollment will occur for eligible patients admitted every day prior from Monday to Friday.
c. Randomization will be performed via the REDCap (Research Electronic Data Capture) system.
d. All eligible patients (both arms) will receive the Best Possible Medication History (BPMH)
e. Patients in the Study arm:
    i. On hospital day 1, or as soon as possible: Receive an Initial Medication Regimen Assessment. Clinical pharmacists assess regimen appropriateness, assess barriers to medication adherence, start filling out the Actions for Assessing Adherence Barriers worksheet
    ii. On hospital day 1, or as soon as possible: Receive a Pre-Discharge Intervention, which, depending on the patient condition, might include a: trial to discontinue certain medications during the hospital; discussion of side effects, flags for new medications prescribed, education about critical barriers to adherence.
    iii. On day of discharge: Receive an At-Discharge Intervention, which may include a brief discussion of medication changes, discussion of side effects, discussion of red flags for new medications. Clinical pharmacists will also consult with inpatient clinicians and pharmacists to discuss the medication list and will ensure that the e-prescribing is done appropriately.
    iv. 1-3 days after discharge: Receive a Post-Discharge Intervention, which includes a call with a patient to discuss the patient's regimen, discussion of side effects, red flags of new medications, a motivational interview, a discussion of barriers to adherence. Other tasks might include a phone call with the patient's primary care physician.
    v. After initial phone call: Some patients, depending on need, might receive a second phone call if needed, a video visit, clinic visit with pharmacist, embedded mental health.
    vi. For any of the above-mentioned activities, patients may be contacted by pharmacists over the phone instead of in-person to reduce COVID-19 exposure to patients, caregivers, and pharmacists.
f. Patients in the Control arm:
    i. Will receive usual care, which may include pharmacist consultation and/or after-discharge phone call(s) if clinically necessary.
g. Critical covariates, including, age, sex, gender, all recorded diagnoses, number of medications, all recorded medication types, zip code, first language, marital status, employment, social history (e.g. tobacco use, alcohol use) will be extracted from electronic health records (EHR) after the intervention ends at each study site. The complete list of data elements to be extracted from the EHR for evaluating trial outcomes are provided in **Appendix A**.

(2) Qualitative study to study barriers and facilitators of implementing PHARM-DC:

a. In the first year of the intervention, the investigators (Dr. Pevnick and Dr. Kennelty) will visit trial sites to conduct 6 focus groups of 6 to 8 clinicians. These focus groups will be grouped by type of clinician (pharmacist, pharmacy tech, physician, nurse). We estimate that 48 people per site will participate in the study.
b. We will use a purposive sampling design by role and department to determine eligibility.
c. Eligible participants will receive an email from study staff inviting them to participate in the focus groups.
    i. Documents:
        1. Recruitment email
        2. Information sheet
d. Focus groups will be 60-90 minutes and will be administered by the PI, the qualitative researcher, and study staff. Due to COVID-19, the focus groups may take place virtually using video conferring software (e.g. Webex).
e. We may also conduct phone or video interviews in lieu of focus groups if individuals are not available for the focus groups.
f. In Years 1-3 of the intervention, Drs. Kennelty, Murry and Keller will conduct 40 pharmacist observations that will last approximately 2 hours each. This may include observations with patients/caregivers, however, the research subject of these observations will be the pharmacist. No PHI or identifiable information will be collected. Researchers will shadow pharmacists for up to 2 hours at a time to understand pharmacist activities and workflow. Observation data will include handwritten notes on either paper or a secure tablet. Examples of observation data include: (1) how pharmacists gather information for medication reconciliation (whether they consult SureScripts, call community pharmacists, call the primary care physician, (2) how they manage doing the intervention when the hospital census is very high, (3) the order in which they do the intervention activities. The observations will be collected using paper and pencil. The observations will take place in the two sites where the pharmacists conduct their regular activities. The pharmacists will be given an updated information sheet (for Study 2) that describes these activities. Patients will be told that this is part of an ongoing study examining pharmacist activities.
g. In Year Two, Dr. Keller will conduct 15 in-depth one-on-one phone interviews with frontline pharmacists asking about the intervention and about challenges pharmacists are having with deprescribing. We estimate that the phone interviews will last 60 minutes.
h. In Year Three, Dr. Kennelty will conduct in-depth one-on-one phone interviews with frontline pharmacists and key stakeholders (e.g., pharmacist leaders). Interviews will last about 25-90 minutes. We estimate that we will interview approximately 50 participants per site.
    i. Documents:
        1. Recruitment email
        2. Information sheet
i. The audiotaped transcripts from both the focus groups and interviews will be transcribed a professional transcribing company. The data will be stripped of identifiers and will be sent to Dr. Kennelty and her staff.
j. The data will be analyzed using NVivo by Dr. Kennelty and study staff.

(3) Cost-Effectiveness Study to analyze the costs of PHARM-DC:

a. Pharmacists will be approached for oral consent at the beginning of a clinic day or clinical shift. Potential participants will be approached, given an information sheet, and the nature of the study will be explained.
    i. Documents:
        1. Information sheet
b. Inclusion Criteria: Pharmacists participating in the PHARM-DC intervention actively seeing patients and responsible for generating the majority of documentation for the clinical visit
c. The observers will use the data collection tool to record time spent on PHARM-DC activities for study patients versus time spent on activities for patients receiving usual care. A trained member of the study staff will shadow participating pharmacists continuously for 4-8 hours during a clinical shift, using an online application (TimeCaT version 3.9, available at lopetegui.net/timecat/39/login/) to note the nature of activities the pharmacist performs and the beginning and ending times of each activity. The TimeCat application requires username and password login, will be installed on a Cedars-Sinai issued iPad, and will only be accessed on the site's secure internet network. The observations will take place in the two sites where the pharmacists conduct their regular activities. Observations may include patients/caregivers. The pharmacists will be given an updated information sheet (for Study 3) that describes these activities. Patients will be told that this is part of an ongoing study examining pharmacist activities.
d. Patients will be assigned a random unique identifier within the TimeCaT application so that no PHI is transmitted over the internet. This will enable the observer to record pharmacist activities performed at the patient level (i.e., per patient). The observer will record the patient MRN, CSN, and unique identifier in a linking file stored separately on Box (https://cedars.app.box.com), along with the number of days since discharge for the relevant patient (e.g., post-discharge day 4).
e. To assure that the time-use data is representative, we will sample approximately 100 patient interactions (20 complete interventions, 20 discharge medication reconciliations, 20 post-discharge phone calls, and 40 observations of usual care) by study pharmacists at each study site over a one-year period.
f. To ensure that pharmacists have become efficient in providing the intervention, sampling will only occur in years two and three and pharmacists with less than two-months experience with the intervention will be excluded.
g. Covariates of patients, including age, sex, gender, race/ethnicity, all recorded diagnoses, number of medications, all recorded medication types, zip code, first language, marital status, employment, social history (e.g. tobacco use, alcohol use) will be extracted from electronic health records after the study period ends at each study site. We will exclude data for patients who have opted out of research studies from data extracts.

2 INTRODUCTION

2.1 STUDY RATIONALE

The sickest patients in the community are recently hospitalized elders. A substantial component of their morbidity and mortality is caused by adverse drug events (ADEs). ADEs account for 70% of adverse events occurring after discharge, which occur at a rate of 0.30 ADEs per patient.[i-iii] The oldest, sickest patients are at highest risk for ADEs: they have the most complex and hazardous medication regimens but the fewest social and economic resources and the least physiologic reserve.[iv] Such patients commonly face challenges in understanding, obtaining, administering, and monitoring new medications prescribed at discharge. These difficulties can lead primarily to avoidable side effects, non-adherence, and suboptimal disease management, and secondarily to outpatient and emergency department visits, hospital readmission, morbidity, and death.[v]

In 2006, one of our investigators published a randomized controlled trial (RCT) showing that pharmacist counseling before discharge and a telephone call after discharge decreased the prevalence of preventable post-discharge ADEs from 11% to 1% (p=0.01).[vi] If implemented nationally, this simple and scalable intervention could prevent 3.5 million post-discharge ADEs annually. Surprisingly, over a decade later, it remains under-resourced and thus underutilized even at the institution where the research was conducted.

Pharmacist leaders around the country report similar implementation difficulties. Many have adopted similar pharmacist discharge care interventions on a limited scale and report anecdotal success from limited use. However, nearly all pharmacist leaders struggle to obtain operational funding to provide this service to all patients likely to benefit. We attribute this struggle in part to a weak business case for hospital leaders to invest in it. The business case is weak because studies so far have looked at abstract concepts like “preventable post-discharge ADEs” rather than healthcare utilization. In annual organizational budgeting, interventions known only to prevent suboptimal *processes* of care are difficult to justify, and thus particularly prone to cuts. As such, we seek to measure the effect of this intervention on utilization reduction, which is the *central innovation of this project.*

2.2 BACKGROUND

Recently hospitalized elders represent the sickest patients in the community. Physiologic reserves, known to lessen with age, are often even lower after hospitalization due to illness, medication, or surgery.[vii] Medication regimens are changed during about half of elderly inpatient hospital stays.[viii] Hospital providers usually increase regimen complexity.[ix] As a result of all this, inappropriate under- and over-prescribing usually increase.[x] Unfortunately, recently hospitalized elders are often unable to fully understand such regimen changes, to obtain the new medications, to advise pharmacists not to fill older prescriptions that have been discontinued, to adhere to new regimens,[xi] or to appropriately monitor themselves for potential side effects. Although seniors’ physiologic reserves may not be remediable, medication regimen appropriateness and adherence can be improved, both of which can lessen rates and severity of adverse drug events (ADEs).

Unsurprisingly, the risks noted above can increase the rate and severity of post-discharge ADEs (defined as any injury due to medication in the 30 days following discharge) among recently hospitalized seniors. In particular, we note:

- Post-discharge ADEs occur in 17-19% of older patients.[xii,15]
- Post-discharge ADEs cause 23-38% of readmissions in older adult patients.[xiii-xv]

Two studies show that most post-discharge ADEs are either preventable (24-27%) or ameliorable (33-38%) (for practicality, these analyses treat the two categories as being mutually exclusive).[xvi,xvii]

Interventions that Reduce Post-Discharge ADEs: Three Major Types (We will Study Two Types)

For clarity, we separate pharmacist-led post-discharge medication management interventions into three major types. From consulting an accepted conceptual framework of medication management,[xviii,xix] we have determined that almost all pharmacist-led interventions reducing post-discharge ADEs do so by addressing medication reconciliation (med rec), medication adherence, or polypharmacy:

1. Med Rec: These interventions seek to find out what medications a patient has been prescribed and has been taking, and to use this information to make sure patients receive correct medications anywhere in the health system.

We believe that the greatest benefit of med rec may be that it *serves as a foundation for* providers to address the two other issues below. Indeed, recent work by ourselves and others to increase implementation and dissemination of med rec is one reason why our proposed RCT is so timely – it is only recently that many patients are now receiving the necessary med rec that will allow them to benefit from interventions targeted at improving medication adherence and reducing inappropriate polypharmacy.

2. Medication Adherence: These interventions seek to improve patients' adherence to a prescribed medication regimen, which may include identifying barriers (e.g., cost, side effects, understanding) and addressing them.

3. Polypharmacy: This refers to the fact that many patients, especially older patients with multimorbidity, are overprescribed medications. It is facilitated by multiple factors, including fragmented care and fear of discontinuing old medications. Many pharmacist-led interventions to reduce polypharmacy involve '*Medication Review*,' wherein pharmacists critically appraise medication regimens and initiate improvements (including addressing under-prescribing, which is now known to also be prevalent in this population).[xx]

We attribute some of the poor uptake of interventions to reduce ADEs to the fact that different interventions come from different disciplines and go by different names. Part of the innovation of our study is that we aggregate potentially synergistic interventions that tend to be based in separate clinical communities: pharmacy (medication review), medication safety (med rec), and geriatrics (polypharmacy).

Pilot Data from Brigham and Women's Hospital (BWH)

Dr. Schnipper's 2006 trial randomized 178 patients being discharged home to either usual care or an intervention with pharmacist counseling at discharge and a follow-up pharmacist telephone call 3-5 days later. Interventions focused on clarifying medication regimens; reviewing indications, directions, and potential side effects; screening for barriers to adherence and early side effects; and providing patient counseling or physician feedback. *This initial study was powered on reducing the rate of preventable post-discharge ADEs.*

Comparing trial outcomes 30 days after discharge, preventable ADEs were detected in 11% of patients in the control group but only in 1% of patients in the intervention group (p = .01).

Despite this success in reducing preventable ADEs, and despite being cited in over 500 publications, this intervention remains underfunded to achieve these outcomes even at Brigham and Women's Hospital (BWH), where the study was conducted. No differences were found in an unpowered comparison between groups in total health care utilization. However

there was a statistically significant decrease in preventable medication-related utilization: only 1 preventable medication-related ED visit or readmission occurred in the intervention group, as opposed to 7 in the control arm (p = 0.03). This suggests the trial was underpowered to detect the effect on utilization.

B.2.2 Pharmacist Intervention to Decrease Medication Errors in Heart Disease Patients (PILL-CVD)[xxi]

This 2-site RCT, for which Dr. Schnipper served as site-PI at BWH, randomized 851 cardiac patients to either usual care or pharmacist-assisted med rec. The pharmacist assistance included inpatient pharmacist counseling, low-literacy adherence aids, and individualized telephone follow-up after discharge. There was no significant change in the primary outcome, the per-patient number of clinically important medication errors (unadjusted incidence rate ratio, 0.92 [95% CI, 0.77-1.10]).

We attribute much of this failure to the use of non-clinician study coordinators rather than pharmacists for phone calls. Especially for the patients targeted by our RCT (taking ≥10 medications or ≥3 high-risk medications, age ≥55), we anticipate complex medication regimens that will require at least two 'touches' by a pharmacist to ensure proper post-discharge medication management. Despite missing its primary outcome, the PILL-CVD intervention reduced early unplanned health-care utilization among patients with inadequate health literacy (aHR 0.41, 95% CI 0.17-1.00).[xxii] Given the results of PILL-CVD, we have taken four major steps to ensure that the best possible version of PHARM-DC is tested on the most appropriate patients in our RCT:

1. Having pharmacists make the post-discharge phone calls and lead other post-discharge activities
2. Providing pharmacists with an *updated evidence-based Toolkit of medication management interventions*
3. Focusing the intervention on high-risk patients: age ≥55, taking ≥3 high-risk medications or ≥10 overall[xxiii,xxiv]
4. Not requiring a cardiac diagnosis (done in PILL-CVD to align with the goals of a funder)

Pilot Data from Cedars-Sinai Medical Center (CSMC)

B.3.1 A Three-Arm Pragmatic Randomized Controlled Trial of Adding Admission Medication History Interviews by Pharmacists or Pharmacist-Supervised Pharmacy Technicians to Usual Care[xxv]

Dr. Pevnick recently completed an RCT that studied med rec in the emergency department. The trial is an example of using pharmacy personnel for medication management. It was successful, reducing admission medication order errors by >80% (3.2 errors per patient in the usual care arm vs only 0.6 per patient in each intervention arm, p<0.0001). The RCT easily enrolled more than 300 patients with a mean age of 72 and a mean of 15 medications over 23 enrollment days, a rate of over 13 daily. This shows that the CSMC team has the operational capacity to enroll a large number of older sicker patients each day. Critically, this not only reflects the

patient population at CSMC but also the aging US population and its increasing polypharmacy.[xxvi]

B.3.2 An Unpowered Nested RCT Suggested Post-Discharge Pharmacist Phone Calls Reduced Readmissions

Analyzing a nested trial within this RCT looked at the effect of post-discharge pharmacist phone calls to improve medication management among patients with suboptimal medication adherence or literacy.[xxvii] Although this unpowered analysis did not yield significant findings, and although there was substantial dropout due to patients not answering phone calls at 30 days, the point estimate for readmissions was 10% lower (absolute drop of 10%) at 30 days in the group receiving phone calls. This shows that CSMC pharmacists have the operational capacity to make post-discharge phone calls, and suggests that the intervention might reduce readmissions, even when pharmacists were not provided with a toolkit of updated evidence-based content.

B.3.3 PHARM-DC Toolkit Development: Results to Date and Work to be Completed in 2017 for ASHP

To identify, aggregate, and synthesize existing evidence regarding the content that pharmacists should provide during the PHARM-DC intervention, we obtained grant-funding from the American Society for Health-System Pharmacists in 2016 to use a literature review and technical expert panel to develop the "PHARM-DC Toolkit." We used an overview of systematic reviews approach[xxviii] (searching MEDLINE, Cochrane and DARE) to identify 74, 14, and 28 existing systematic reviews in the areas of medication adherence, polypharmacy, and post-discharge med rec, respectively. In cases where we found at least one high quality systematic review (AMSTAR $\geq$8)[xxix] with reasonable evidence that an intervention could successfully improve even intermediate processes demonstrating improved post-discharge medication management, we have traced the literature findings back to the original paper or best summary of the intervention for inclusion in the PHARM-DC toolkit.

2.3 RISK/BENEFIT ASSESSMENT

2.3.1 KNOWN POTENTIAL RISKS

Hospital Discharge Study

This is a low-risk intervention. Since the intervention is already being conducted at the two sites by clinical pharmacists when there is administrative capacity to do so, we do not anticipate any additional risks involved with the hospital discharge intervention.

There is a small risk of breach of confidentiality with regards to the electronic health records data collected by the researchers at the end of the study; however, all data will be kept on password-protected, encrypted study site computers. Only investigators and study staff who are IRB-approved will have access to the study data. Since we are examining data at the end of the intervention, the data analysis is similar to a retrospective secondary data analysis.

To reduce the risk of COVID-19 exposure, some or all of the intervention activities (medication reconciliation, patient/caregiver education and counseling) will be conducted over the phone.

Focus Groups and Interviews

Individuals who participate in the focus groups and/or interviews may experience mild discomfort about certain questions. However, they will be reminded at the beginning of the focus group that they are not required to answer any questions they find cause discomfort and that they may stop the focus group/interview at any time.

There is also a small risk of breach of confidentiality. Interviewees/participants of focus groups/interviews will be given number identifiers, which will be kept separate from their identifiers/demographic information. Only the study staff will have access to the identifiers. This sub-study will not include any patient observation or use of PHI.

Observations will not affect any clinical decisions or clinical care. The pharmacist will be notified that observations will take place and that no identifiable information will be recorded about the interactions. Patients who may have interactions with the pharmacists will not be notified of the observations for research, since information will not be collected about them. Individuals who participate in the observations may experience mild discomfort when being observed. However, they will be reminded at the beginning of the observation that they are not required to participate in the observations and can stop the observation at any time. There is also a small risk of breach of confidentiality. Participants in the observations will be given number identifiers, which will be kept separate from their identifiers/demographic information. Only the study staff will have access to the identifiers.

Time and Motion Study/Cost-Effectiveness Analysis

There is a small risk of breach of confidentiality. Patients will be assigned a unique identifier within the TimeCaT application so that no PHI is transmitted over the internet. Only study staff will have access to the identifier and linking file, which will be stored separately on Box and will only be accessed on a password protected, encrypted study site computer. When not in use, iPads will be stored in a locked cabinet behind a locked office door in the Department of Medicine's administrative office at each site.

Observations will not affect any clinical decisions or clinical care. The pharmacist will be notified that observations will take place. Patients who may have interactions with the pharmacists will be told that this is part of an ongoing study examining pharmacist activities. Individuals who participate in the observations may experience mild discomfort when being observed. However, they will be reminded at the beginning of the observation that they are not required to participate in the observations and can stop the observation at any time.

There is also a small risk of breach of confidentiality with regards to the electronic health records data collected by the researchers at the end of the study; however, all data will be kept on password-protected, encrypted study site computers. Only investigators and study staff who are IRB-approved will have access to the study data. Since we are examining data after the end of the study period at both sites, the data analysis is similar to a retrospective secondary data analysis. We will exclude data for patients who have opted out of research studies from data extracts.

2.3.2 KNOWN POTENTIAL BENEFITS

Individuals may receive consultations with pharmacists that help them understand their medications and/or chronic conditions more thoroughly.

3 OBJECTIVES AND ENDPOINTS

Primary Outcome

We chose an endpoint of 30-day post-discharge utilization as our primary outcome because the RE-AIM model points to the importance of institutional-level effectiveness and cost. We chose to power this trial on an absolute utilization reduction of 2.5% (% of patients with readmissions or ED visits within 30 days of hospital discharge) from a baseline of 27.5%.

We define this utilization in two different ways, based on what data is available. For the first analysis, we will use same-hospital utilization data. Both site PIs can electronically access inpatient admission, observation stay, and ED visit data for their health system. These three utilization types will represent the basis for the first RCT analysis (*manuscript #2 above)*.

We recognize the limitations of same-hospital utilization data.[xxx] As such, the RCT is powered on a *primary endpoint of same-state 30-day hospital readmissions and ED visits* *(manuscript #6 above*).

This utilization will be tracked with data from the Massachusetts All Payer Claims Data (APCD) database and California's Office of Statewide Health Planning and Development.[xxxi] Because we expect little utilization in other states (discharge to another state is an exclusion criterion) and because we do not anticipate any differential effect on such utilization, we will not track it. Statewide data sources do not track observation stays, so they will be excluded from statewide analyses. However, we will conduct a sensitivity analysis projecting changes in same-hospital observation stays to the statewide analysis.

The current version of this protocol does not include information for these analyses using other data sources; we will include this information in subsequent versions through protocol/IRB amendments.

Secondary Outcomes

We will examine the endpoints in the following subgroups:

Patients:
1) Patient socioeconomic status (estimated via median income of home census tract);
2) Patient medication adherence and literacy (as assessed by study pharmacists, in addition to using limited English proficiency as a proxy for low medication literacy);
3) Study site;
4) Ten or more medications;
5) Three or more high risk medications;
6) Patient history of congestive heart failure;
7) By receipt of different intervention components;
8) Patient age groups;
9) Before and after historical changes to the intervention and control arms;
10) Primary outcome restriction to medication-related post-discharge utilization;
11) Primary outcome restriction to 7-day post-discharge utilization.
12) 30-day mortality
13) Effect of the intervention on each type of utilization considered separately (ED visits alone, observations stays alone, readmissions alone)

4 STUDY DESIGN

4.1 OVERALL DESIGN

Hospital Discharge Study

This is a prospective, randomized, controlled, pragmatic trial of a pharmacist-led intervention aimed at reducing readmissions. The study will focus on discharge utilization among patients most at risk for post-discharge ADEs: recently discharged older adults taking $\geq$10 medications or $\geq$3 high-risk medications.

Focus Groups and Interviews

This is a qualitative study employing interviews, observations, and focus groups aimed at understanding implementation barriers and facilitators. Drs. Pevnick and Kennelty and study staff will conduct 3-day site visits to hold focus groups regarding intervention expectations, how the intervention is being implemented, barriers/facilitators to adoption and implementation, and adaptations made during delivery.

Towards the end of the trial, Dr. Kennelty and study staff will use phone interviews to determine any changes to the implementation of PHARM-DC that occurred during the RCT, as well as to understand whether and how institutions will address the maintenance of PHARM-DC after the study ends.

We will use qualitative methods to the reach of the intervention (overview in **Table 6** above), and to investigate several hypotheses developed using RE-AIM as a guide.

<u>We hypothesize that *staff adoption* will be facilitated by</u>:

(1) units and sites with better safety culture and teamwork and

(2) unit-based (vs central) pharmacists, whereas <u>barriers to *staff adoption* will include</u>:

> (1) difficulties associated with coordinating a time for discharge counseling (patients are frequently anxious to leave as soon as discharge orders are placed, but the discharge medication regimen is not usually available before this time), and
> (2) that even when problems are identified, lack of resources may still be an issue (e.g., medications with expensive copayments). <u>We hypothesize that *implementation consistency* will be higher and that *adaptations will be lower*</u> when pharmacists are not overwhelmed by other responsibilities, and on medical (vs surgical) units.

<u>Surveys to Assess Organizational Context:</u> We will assess safety culture with the AHRQ Patient Safety Survey. Individuals who participate in the focus groups/interviews will be asked to complete the survey prior to participating in the study.

The observations will take place at the two study sites (CSMC and BWH). They may include observations of patients/caregiver interactions. Researchers will not collect any PHI. Observations will include the collection of information such as:

- Amount of time spent by pharmacists on each individual task
- Challenges pharmacists have when attempting to contact caregivers, nurses, pharmacist technicians, primary care physicians, and attending physicians
- Number of sources that pharmacists use (SureScripts, retail pharmacies, asking caregivers) to put together medication reconciliation information
- Challenges pharmacists have when trying to contact patients (e.g. patient doesn't answer the phone)
- How pharmacists divide up work when there are a lot of patients in the study (e.g. how they each have a floor to cover and how they cover for each other when one is busy or is out).

Time and Motion Study

This is a time-and-motion study aimed at understanding how much time during the day pharmacists spend on activities related to the hospital discharge (and post-discharge) interventions. During the trial, we will use time-and-motion methodology to understand how much pharmacist time is needed for PHARM-DC. Coupled with data on post-discharge utilization, this will enable us to conduct economic analyses.

Researchers will collect pharmacist demographics, including:

1. Name
2. Study site
3. Years of experience as a pharmacist
4. Years of experience in TOC
5. Years of Experience in Hospital-Based Pharmacy
6. Years of Residency Training
7. Month and year that you started working at the study site
8. Month and year that you started delivering the PHARM-DC intervention
9. Type of Residency Training
10. List of Board Certifications and Credentials

5 STUDY POPULATION

5.1 INCLUSION CRITERIA

Inclusion criteria include:

- Being discharged from a medical ward, **AND**
- $\geq$ 55 years old **AND**
- ≥ 10 chronic prescription medications

OR

- $\geq$ 3 high-risk medications (anticoagulants, antiplatelets, insulin, oral hypoglycemics, and) before or during hospitalization

5.2 EXCLUSION CRITERIA

Exclusion criteria include the following:

- Expected discharge to another state, acute care facility, psychiatric facility, or locked facility (including locked skilled nursing facility, jail, or prison) **OR**
- Expected leaving hospital against medical advice (AMA) or actual AMA **OR**
- Homeless **OR**
- On hospice **OR**
- Already enrolled into study during prior discharge in previous year **OR**
- Expected to receive pharmacist-led post-discharge medication management regardless of the trial **OR**
- Patients admitted by admitting or attending Primary Medical Doctors who have a specialty that is not Internal Medicine or Family Medicine **OR**
- Expected post-discharge setting not conducive to the studied medication management intervention **OR**
- Patients admitted with a suspected or confirmed diagnosis of COVID-19.

5.3 STRATEGIES FOR RECRUITMENT AND RETENTION

Hospital Discharge Study

Eligible pharmacists will be selected by the pharmacy managers at each site. Eligibility includes training in transitions of care and/or post-discharge care.

A daily Epic report will be generated at each site to identify eligible patients. A research assistant will conduct a brief chart review to confirm eligibility before randomizing the patient using REDCap. For quality assurance, operational reports will be obtained from the emergency department and cross-checked with the EIS report to ensure all patients who may be eligible for the study are accurately identified. The study coordinator will stop randomizing patients if the number of patients concurrently receiving the intervention on any given weekday exceeds the workload capacity of pharmacists at each site. Study coordinators will communicate with pharmacists to regularly re-assess the maximum threshold of concurrent patients in the intervention arm. This strategy aims to reduce the potential for pharmacist burnout and to optimize intervention quality.

Once the patient has been randomized to the intervention or control arm, the clinical pharmacist will approach the patients in the intervention arm.

Focus Group/Interviews Study

We will use a purposive sampling design by role and department to determine eligibility. Eligible participants will receive an email from study staff inviting them to participate in the focus groups, and/or interviews. To enhance recruitment, we will offer a $125 honorarium to nurses and a $250 honorarium to physicians for their participation in a 60-90 minute focus group or interview.

Time and Motion Study

Pharmacists will be approached for oral consent at the beginning of a clinic day or clinical shift. Potential participants will be approached, given an information sheet, and the nature of the study will be explained. Inclusion Criteria: Pharmacists participating in the PHARM-DC intervention actively seeing patients and responsible for generating the majority of documentation for the clinical visit.

6 STUDY INTERVENTION

6.1 STUDY INTERVENTION(S) ADMINISTRATION

6.1.1 STUDY INTERVENTION DESCRIPTION

PHARM-DC Intervention Overview

Pharmacists at both study sites have experience in providing discharge counseling and in making post-discharge phone calls. This experience will ensure that PHARM-DC is being tested in its best light. The Toolkit content and 1-2 hours of introductory training add three major components:

1. Rather than incorporating content based solely upon their prior training and experience, pharmacists will use the *PHARM-DC Toolkit,* which *summarizes the evidence* for post-discharge medication management.

2. Pharmacists will *assess patient needs* and *customize accordingly*. All patients will receive one discharge counseling visit and one post-discharge phone call, but pharmacists will find that some patients need further phone calls, interventions, referrals, or other interactions.

3. Although pharmacists have long been successful in leading med rec interventions, most work focused on *what medications patients had been prescribed and are taking*. This is a necessary step, but we believe most benefit occurs from the subsequent step of figuring out what medications patients *should be* taking.

PHARM-DC toolkit:

1) Pharmacist review of discharge med rec and medication appropriateness (e.g., reduction in polypharmacy);

2) In-hospital room visit to screen for previous barriers to safe medication use, initiate management of these barriers, and communicate with post-discharge providers regarding likely actions that will need to be taken;

3) Post-discharge phone call by a pharmacist 1-3 days after discharge to screen for patient/caregiver lack of understanding of their medication regimen, medication non-adherence, early side-effects, lack of knowledge of what ADEs to watch for and what to do if they occur, other barriers to safe medication use, and management of these problems;

4) Based on this risk assessment, pharmacists will tailor the interventions to each patient. The personalized intervention may include:

   a. ***Patient education, counseling, and coaching*** regarding the importance of taking medication as prescribed and contacting a designated caregiver or provider if there are problems;
   b. ***Taking further steps to improve medication appropriateness;***
   c. ***Post-discharge assistance with obtaining medications,*** including drug assistance programs (for reduced pricing) and linkage with community or social service organizations;
   d. ***Pharmacist communication with key inpatient and outpatient providers*** (e.g., PCPs; skilled nursing facilities; inpatient rehabilitation hospitals, including the California Rehabilitation Institute) to ensure that they understand changes to their patient's discharge medication regimen, including why those changes were made and recommended actions to take. This includes requesting Medication Administration Records (MAR) from downstream providers to facilitate this type of communication;
   e. ***Communication with each patient's pharmacy*** to ensure the pharmacy has an updated medication list, with focus on newly discontinued medications, and to encourage patient education as needed when patients fill their prescriptions;
   f. ***Use of illustrated pill cards, pillboxes (traditional and electronic), and other tools*** to help patients with low literacy better understand and adhere to their medication regimens;
   g. ***Health information technology such as patient portals and texting*** chatbots to facilitate communication between patients, pharmacists, and other providers after discharge;
   h. ***Home pharmacist visits for high-risk patients*** to educate them and to identify and manage challenges to safe medication use.

6.2 MEASURES TO MINIMIZE BIAS: RANDOMIZATION AND BLINDING

Randomization will be performed via the REDCap (Research Electronic Data Capture) system. REDCap is a secure, web-based application supporting data capture for research studies, providing:

1) an intuitive interface for validated data entry;
2) audit trails for tracking data manipulation and export procedures;
3) automated export procedures for seamless data downloads to common statistical packages; and;
4) procedures for importing data from external sources.

REDCap will incorporate allocation tables generated in R. Study participants will be randomized 1:1 to the two arms of the study and stratified by site. Arm assignment will not be actively masked, but most clinical personnel and patients will remain unaware of the trial, just as they are unaware of the details of most post-discharge medication management practices currently.

6.3 STUDY INTERVENTION COMPLIANCE

We will use EHR documentation to assess study intervention compliance. This will include detailed reports generated from Epic about pharmacist documentation notes and phone calls.

7 STUDY INTERVENTION DISCONTINUATION AND PARTICIPANT DISCONTINUATION/WITHDRAWAL

7.1 LOST TO FOLLOW-UP

Hospital Discharge Study

Patients who leave the state or country after their hospital discharge or who do not answer phone calls post-discharge will be considered lost to follow-up and this will be recorded in the CONSORT diagram.

7.2 ADVERSE EVENTS AND SERIOUS ADVERSE EVENTS

7.2.1 DEFINITION OF ADVERSE EVENTS (AE)

Adverse event means any untoward medical occurrence associated with the use of an intervention in humans, whether or not considered intervention-related (21 CFR 312.32 (a)).

7.2.2 DEFINITION OF SERIOUS ADVERSE EVENTS (SAE)

An adverse event (AE) or suspected adverse reaction is considered "serious" if, in the view of either the investigator or sponsor, it results in any of the following outcomes: death, a life-threatening adverse event, inpatient hospitalization or prolongation of existing hospitalization, a persistent or significant incapacity or substantial disruption of the ability to conduct normal life functions, or a congenital anomaly/birth defect. Important medical events that may not result in death, be life-threatening, or require hospitalization may be considered serious when, based upon appropriate medical judgment, they may jeopardize the participant and may require medical or surgical intervention to prevent one of the outcomes listed in this definition. Examples of such medical events include allergic bronchospasm requiring intensive treatment in an emergency room or at home, blood dyscrasias or convulsions that do not result in inpatient hospitalization, or the development of drug dependency or drug abuse.

7.2.3 CLASSIFICATION OF AN ADVERSE EVENT

7.2.3.1 RELATIONSHIP TO STUDY INTERVENTION

We do not anticipate any adverse events related directly to the study intervention. Therefore, AEs will be classified as:

Not Related – There is not a reasonable possibility that the administration of the study intervention caused the event, there is no temporal relationship between the study intervention and event onset, or an alternate etiology has been established.

Each site PI, Dr. Josh Pevnick (CSMC) and Dr. Jeffrey Schnipper (BWH) will be responsible for determining whether an adverse event (AE) is expected or unexpected. An AE will be considered unexpected if the nature, severity, or frequency of the event is not consistent with the risk information previously described for the study intervention. AEs will be reported to the DSMB for evaluation.

7.2.4 TIME PERIOD AND FREQUENCY FOR EVENT ASSESSMENT AND FOLLOW-UP

The occurrence of an adverse event (AE) or serious adverse event (SAE) may come to the attention of study personnel during study visits and interviews of a study participant presenting for medical care, or upon review by a study monitor. Study staff will immediately notify the principal investigator (Dr. Josh Pevnick) upon becoming aware of deaths occurring in study participants. The PI will report deaths to the NIA PO within 48 hours by email and submitting the appropriate case report form (CRF).

Deaths and same-hospital readmissions (inpatient, observation, or emergency department visits) occurring within 90-days of hospital discharge will be collected retrospectively from the patient's medical record every six months through the study. All AEs/SAEs will be reported in a semi-annual summary safety report to the Data Safety Monitoring Board (see Section 9.1.4).

All AEs including local and systemic reactions not meeting the criteria for SAEs will be captured on the appropriate CRF. Information to be collected includes event description, time of onset, clinician's assessment of severity, relationship to study product (assessed only by those with the training and authority to make a diagnosis), and time of resolution/stabilization of the event. All AEs occurring while on study must be documented appropriately regardless of relationship. All AEs will be followed to adequate resolution.

Any medical condition that is present at the time that the participant is screened will be considered as baseline and not reported as an AE. However, if the study participant's condition deteriorates at any time during the study, it will be recorded as an AE.

Changes in the severity of an AE will be documented to allow an assessment of the duration of the event at each level of severity to be performed. AEs characterized as intermittent require documentation of onset and duration of each episode.

8 STATISTICAL CONSIDERATIONS

8.1 STATISTICAL HYPOTHESES

Hypothesis 1: PHARM-DC will reduce post-discharge utilization reduction of 2.5% (% of patients with readmissions or ED visits within 30 days of hospital discharge) from a baseline of 27.5%.

8.2 SAMPLE SIZE DETERMINATION

We will use a two-sided likelihood ratio test to compare the proportion of patients with utilization within 30 days of discharge between the intervention and usual care groups. Assuming 80% power and a type I error rate of 0.05, a two-sided Z test between two proportions with **4,888 subjects per group** would detect a difference of at least 2.49% from a baseline of 27.5%. Thus, our primary statistical analysis will be a two-sided Z test. We will also compare the baseline characteristics of those subjects across both arms to ensure there are no major differences. All enrolled patients will be analyzed.

Table 5 shows that varying the baseline utilization proportion does not substantially alter the minimum detectable difference. We also performed a sensitivity analysis assuming that 7.5% of patients in the intervention arm were unreachable or refused a phone call. Even at this rate, which is our best estimate from our pilot data, we would have 80% power to detect a difference of 2.54%. Finally, these rates were robust to contamination rates of 2% among patients in the usual care arm. We believe this low rate is a reasonable estimate for contamination, because patients thought to have a clear need for these services will be excluded from the RCT, and because we believe that non-pharmacist clinicians will remain so busy that they will not begin to provide such services to other patients.

Table 5. How Baseline Utilization Affects Minimum Detectable Difference

Percentage of Patients with Utilization within 30 Days of Hospital Discharge		
Usual Care	Intervention	Minimum detectable difference
30.0%	27.44%	2.56%
27.5%	25.01%	2.49%
25.0%	22.59%	2.41%

For the qualitative study, the study will enroll up to 48 people in the focus groups, 15 people in the Year 2 interviews, and 50 people in the interviews per site.

8.3 POPULATIONS FOR ANALYSES

We will conduct the following analyses:

- Intention-to-Treat (ITT) Analysis Dataset (i.e., all randomized participants)
- Modified Intention-to-Treat Analysis Dataset (e.g., participants who had the in-hospital intervention but did not respond to post-discharge phone calls)
- Per-Protocol Analysis Dataset: defines a subset of the participants in the full analysis (ITT) set who complied with the protocol sufficiently to ensure that these data would be likely to represent the effects of study intervention according to the underlying scientific model (e.g. participants who had both the in-hospital intervention and the post-discharge phone call).

8.4 STATISTICAL ANALYSES

8.4.1 GENERAL APPROACH

- We will use chi-square tests, Analysis of Variance (ANOVAs), logistic regression, and means/medians for descriptive statistics.

- We will use a 0.05 cutoff for p-values and 95% confidence intervals for statistical significance.
- Our primary statistical analysis will be a two-sided Z test. We will also compare the baseline characteristics of those subjects across both arms to ensure there are no major differences. All enrolled patients will be analyzed.
- We will check for normality and log-transform variables if necessary for skewed distributions.
- We will calculate all-cause unplanned post-discharge utilization within a 30-day period from the date of discharge from an index admission. Post-discharge utilization will include emergency department visits, observation stays, and inpatient readmissions. Planned readmissions will be excluded using the Centers for Medicare and Medicaid's Planned Readmissions Algorithm. The algorithm assesses a dichotomous yes or no outcome of whether each admitted patient has any unplanned readmission within 30 days. In accordance with the CMS algorithm, if a patient has more than one unplanned admission within 30 days of discharge from the index admission, only the first is considered a readmission. If the first readmission after discharge is planned, any subsequent unplanned readmission is not considered in the outcome for that index admission because the unplanned readmission could be related to care provided during the intervening planned readmission rather than during the index admission.

8.4.2 ANALYSIS OF THE SECONDARY ENDPOINT(S)

To assess the *Reach* of the intervention, we will examine differences in effectiveness across sites, and for patients of different ages, sex, medication literacy, taking different numbers of medications, and living in zip codes reflecting different median incomes. We hypothesize that the intervention will work best to prevent readmissions, will work equally at both sites, will work better for older patients, those with higher medication literacy, those taking more medications, and those from poorer zip codes. We will also explore effectiveness of the intervention before and after historical changes to the intervention and control groups, as well as when the primary outcome is restricted to include only medication-related post-discharge utilization or 7-day post-discharge utilization. However, we recognize that these underpowered analyses may be most useful for shaping future hypotheses. To ensure that no results are misinterpreted, we will report that any negative results may be due to inadequate power.

The descriptive phase of analysis will include an assessment of the distributions and correlations of the aforementioned variables of interest. We will use logistic regression to examine these factors. We will also examine the potential interaction of covariates. We will explore collinearity using the condition index and with careful assessment of the correlation matrix.

8.4.3 QUALITATIVE ANALYSES

For the qualitative analysis of interviews and focus groups, we will combine a content-analysis approach with qualitative inquiry allowing us to discover and quantify key stakeholders' experiences and perceptions. We will use an iterative process to identify "a priori" themes based on the domains in the RE-AIM framework and to create "in vivo" themes as they emerge during coding (e.g., specific barriers to PHARM-DC implementation).[xxxii] Two coders will code each

interview independently and then discuss variations until consensus is reached. After coding all interviews, we will use the constant comparative method to combine similar themes with limited data under more general themes.[110] We will use NVivo software which will allow for quantifying the number of key stakeholders who addressed these themes and their density (i.e., number of overall mentions). Theme density is a valid proxy for importance. In the final step of the qualitative analysis, we will prepare NVivo code reports (participant N and density) of salient themes and coding matrices related to significant interview findings. We will perform follow-up interviews as needed to supplement or clarify themes. We will also prepare descriptive statistics of all interview and focus group participants.

We will take several measures to ensure rigorous analysis. All transcripts will be independently analyzed by Dr. Kennelty and two other researchers who will meet weekly during the qualitative analysis process to discuss each transcript and resolve disagreements through negotiated consensus.[xxxiii] Analysts will also maintain an audit trail throughout this process to document the fit between the raw data and the conclusions drawn. NVivo software will be used to code patterns among sites and individuals, and to code variations within and between key stakeholders. We will later triangulate survey results with interviews, focus groups, and on-site observations to better understand the impact of context on the experiences of key stakeholders.

Finally, after conducting initial qualitative and quantitative analyses, we will use a mixed methods approach to further explore relationships between contextual factors, interventions, and outcomes.[xxxiv] Qualitative and mixed methods may be especially important if there are any differences between the two sites RCT outcomes and to develop hypotheses regarding possible reasons for our findings.

8.4.4 COST-EFFECTIVENESS ANALYSES

Costs are a critical consideration for health system leaders deciding to adopt an intervention, as noted by the RE-AIM model. Because the leadership at different hospitals may consider different factors when deciding whether to fund the PHARM-DC intervention, we will report the cost of implementing the PHARM-DC intervention in two ways: (1) the program costs per utilization event (readmission, observation stay, or ED visit) averted and (2) the incremental net cost from the health system perspective.

Program Cost: The primary program cost will be the ongoing labor of the pharmacists who implement it. Startup costs will be minimal because hospital pharmacists can be trained to use the PHARM-DC Toolkit in only 1-2 hours, and no purchases of equipment or supplies are needed.

We will ascertain pharmacist labor costs through a micro-costing approach employing accepted time and motion methodology used in our previous work.[xxxv-xxxix] Our published data collection tool, which was developed for admission medication histories, categorizes time spent on discussions with patients, family members, and caregivers, utilizing secondary resources (obtaining prior prescription and fill information), and utilizing the electronic health record to obtain, document, and communicate information.[xl] We will add categories pertinent to post-discharge medication management (e.g., calling retail pharmacies to communicate that a medication has been stopped) and pilot the tool until all relevant activities have been categorized.

Trained observers with a clinical background (e.g., research nurses, medical students) will document time use by study pharmacists on a minute-by-minute basis in real time. The

observers will use the time-use data collection tool to record time spent on PHARM-DC activities for study patients versus other activities. For PHARM-DC activities, the observer will also record the number of days since discharge for the relevant patient (e.g., post-discharge day 4). Because pharmacists will also complete non-intervention tasks each day, observers will follow pharmacists for up to an entire workday.

To assure that the time-use data is representative, we will sample approximately 100 patient interactions (20 complete interventions, 20 discharge medication reconciliations, 20 post-discharge phone calls, and 40 observations of usual care) by study pharmacists at each study site over a one-year period. To ensure that pharmacists have become efficient in providing the intervention, sampling will only occur in years two and three and pharmacists with less than two-months experience with the intervention will be excluded.

Time spent on PHARM-DC activities for study subjects by the number of days since discharge. For example, we will identify all study subjects for which study pharmacists spent time on post-discharge day 1, etc. This will enable us to calculate the mean amount of pharmacist time necessary to provide the PHARM-DC intervention on each post-discharge day, and then the mean total time across all days. After estimating the mean total time per patient, we will apply National Bureau of Labor Statistics wage and benefit data to calculate the intervention's program cost per patient[2].

Next, we will calculate the mean program cost necessary to avert each measured utilization event (e.g., readmission) by multiplying the number needed to treat to prevent the utilization event by the cost of applying the intervention to one patient. For some hospital decision makers, we expect this data, in combination with hospital-specific costs, to be useful in deciding whether (and to what extent) to implement PHARM-DC. One example of hospital-specific costs would be anticipated HRRP costs (CMS readmissions penalties).

Costs of Post-Discharge Utilization: We will ascertain the total cost of all readmissions, observation stays, and ED visits with 30 days of discharge for all trial patients. At least 80% of patients are likely to return to the study hospitals; in this case, we will obtain charge data for the return visits and apply hospital-specific cost-to-charge ratios provided by hospital administrators. For return visits to outside hospitals, we will obtain charges for inpatient hospitalizations and ED visits using statewide data sets and calculate costs by applying cost-to-charge ratios for hospital services published by the Centers for Medicare and Medicaid Services.

Net Costs: Next, we will calculate net cost for each patient from the health system perspective by subtracting the cost of any post-discharge utilization from any program costs (program costs are zero in the control group). Because we will apply a 30-day time horizon, discounting will not be used. We will calculate and test differences in mean values. Finally, we will calculate the difference between the intervention and control group to estimate incremental net costs per patient from the health system perspective.

8.4.5 SECONDARY DATA ANALYSIS: EXAMINE EFFECTS OF PHARM-DC INTERVENTION ON INITIATION AND DEPRESCRIBING OF BENZODIAZEPINE-RECEPTOR AGONISTS

The following secondary analyses of trial data are planned to examine effects of the PHARM-DC intervention on (1) the initiation of new outpatient prescriptions of benzodiazepine-receptor agonists (BZRs) and (2) the deprescribing of BZRs among patients taking BZRs prior to hospitalization:

1. Among patients <u>without</u> a BZR prescription on admission, we will examine the difference in adjusted predicted probability of having a new BZR prescriptions from admission to discharge between the intervention group and the control group. We will use logistic regression using a generalized estimating equations approach and estimate adjusted predicted probabilities.[3] We hypothesize that among patients without a BZR prior to hospitalization, the risk-adjusted probability of having a new discharge BZR prescription will be substantially lower (e.g. 35% lower) among patients who are enrolled in the PHARM-DC intervention compared to the control group.

2. Among patients <u>taking</u> BZRs prior to hospitalization, we will use logistic regression to estimate the adjusted predicted probability of dose intensification among patients in the PHARM-DC intervention and the control group.[3] We hypothesize that among patients with BZRs prior to hospitalization, the risk-adjusted probability of experiencing a dose increase will be substantially lower (e.g. 35% lower) among patients who are enrolled in the PHARM-DC intervention compared to controls.

<u>Outcome measures</u>: We will create two measures: (1) a dichotomous variable for a new discharge prescription of BZRs among patients not taking BZRs prior to admission, and (2) a dichotomous variable for an increased dose prescription among patients taking BZRs prior to admission.

8.4.6 SECONDARY DATA ANALYSIS: EXAMINE EFFECTS OF PHARM-DC INTERVENTION ON FALL-RELATED INJURIES AMONG OLDER ADULTS WITH ALZHEIMER'S DISEASE AND RELATED DEMENTIAS

The following secondary analyses of trial data are planned to examine effects of the PHARM-DC intervention on post-hospitalization fall-related injuries among all trial participants and among the subgroup of older adults with Alzheimer's disease and related dementias (ADRD):

1. We will investigate whether the intervention reduces the <u>severity</u> of fall-related injuries by using a two-sided Z test to compare the proportions of severe injuries (e.g., hip fractures) and acute care utilization (hospitalization and ED visits) between the intervention and control groups. We hypothesize that the PHARM-DC intervention reduces the severity of fall-related injuries by mitigating medication-related risk factors.

2. We will use a two-sided Z test to compare the proportion of those who have post-hospitalization healthcare utilization for fall-related injuries within 30 days among all trial participants and among individuals with ADRD. We hypothesize that the PHARM-DC intervention reduces fall-related injuries among individuals with ADRD. We also hypothesize that the benefit of the intervention is larger among individuals with ADRD compared to

individuals without ADRD by addressing both general and ADRD-specific medication-related risk factors for falls.

3. We will examine the effect of the intervention among individuals with dementia with Lewy bodies (ICD-10-CM code: G31.83) and individuals with an ADRD diagnosis code associated with the ICD-10-CM code for Parkinson's disease (G20), because previous literature suggests that they have a much higher incidence of fall-related injuries, as compared to individuals with Alzheimer's disease.[4]

4. We will describe the difference in the nature of falls among individuals with and without ADRD using external causes of morbidity codes (e.g., falls from bed vs. falls while walking).

Variables: We will check for balanced demographics and comorbidities across arms in all analyses. In cases with imbalance, we will consider post-hoc adjustment, including by considering sex as a biological variable. Individuals with ADRD will be identified by the ICD-10-CM codes based on hospital claims, adapting the algorithm from the Chronic Conditions Data Warehouse.[5]

Outcome measures: We define post-hospitalization fall-related injuries as healthcare utilization (hospitalization, ED visits, urgent care visits, or office visits) due to fall-related injuries within 30 days of discharge of the index admission. We will link Medicare claims data to trial data to track this outcome at the individual patient level. In order to link Medicare claims data to trial data, linking variables for trial participants will be requested through the Honest Enterprise Research Broker (HERB) Committee at Cedars-Sinai and will include: (1) social security numbers (SSNs), (2) Medicare Beneficiary Identifiers (MBIs), and (3) Medicare Health Insurance Claim Numbers (HICNs). We anticipate data may be missing on linking variables for some participants (e.g., patients without an SSN number), and therefore, will request patient age, SSNs, MBIs, and HICNs to ensure data for all participants can be linked to claims data. The same linking variables in (1)-(3) will be requested from the Brigham and Women's Hospital site and uploaded to a password-protected Box folder hosted on the Cedars-Sinai network (for further details, see section 9.1.5.1 DATA COLLECTION AND MANAGEMENT RESPONSIBILITIES). Linking variables for participants at both study sites will be mailed on a flash drive to the Centers for Medicare & Medicaid Services (CMS) based on their submission requirements for requesting claims data. Medicare claims data will subsequently be sent from CMS to the study team at Cedars-Sinai on a flash drive through postal mail.

Sensitivity analysis: It is possible that we under-detect individuals with ADRD diagnoses if the ADRD diagnoses are not coded diligently on hospital claims. To mitigate this issue, we will conduct a sensitivity analysis by using electronic health record (EHR) data prior to the index admission, so that we can include those who were diagnosed with ADRD in the outpatient setting. Because this can only be done for patients who receive outpatient care from doctors using our EHR, it would introduce bias if used as part of the overall analysis. However, it can be done among this group to test the sensitivity of detecting ADRD via claims.

8.4.7 SECONDARY DATA ANALYSIS: PROPENSITY SCORE ANALYSIS AT BRIGHAM AND WOMEN'S HOSPITAL

Admission medication reconcilitation is theorized to be one of the key components of the PHARM-DC intervention that may contribute to reduced 30-day post-discharge utilization. At the Brigham and Women's Hospital (BWH) site, many more patients were eligible for enrollment in the PHARM-DC clinical trial than were actually enrolled due to limited pharmacist staff

bandwidth. For this reason, patients who were eligible, yet were not enrolled, did not receive admission medication reconcilitation (also known as a “Best Possible Medication History” [BPMH] in the literature).

As a secondary analysis of trial data, we propose to compare patients who were eligible for trial enrollment *and* enrolled to patients who were eligible ***and not*** enrolled at BWH. We will perform a propensity score analysis to predict enrollment into the PHARM-DC clinical trial at the individual patient-level. Resulting propensity scores will then be used to identify matches between patients at BWH who were eligible and enrolled vs. eligible and not enrolled during the study period for comparison. The outcome of interest is 30-day post-discharge utilization (same hospital).

Data will be retrieved retrospectively from the electronic health record at BWH as previously described in this IRB protocol. Specifically, variables in the propensity model will include:

1. Time of day of hospital admission
2. Day of week of hospital admission
3. Day of week of hospital discharge
4. Admit type
5. Admit service
6. Discharge service
7. Hospital length of stay
8. Discharge location
9. Admissions medications list
10. High-risk medications list at admission
11. Calendar quarter
12. Insurance type
13. Admission source
14. Interaction effect between length of stay *and* day of week of admission

9 SUPPORTING DOCUMENTATION AND OPERATIONAL CONSIDERATIONS

9.1 REGULATORY, ETHICAL, AND STUDY OVERSIGHT CONSIDERATIONS

9.1.1 INFORMED CONSENT PROCESS

Hospital Discharge Study

Justification for Randomization of the PHARM-DC Intervention with a Waiver of Informed Consent

As noted above, both sites already conduct the majority of components of the PHARM-DC intervention on some patients. Because it is currently used, but allocation is determined by operational convenience (e.g., pharmacists try to provide this service to everyone they think would benefit, but only reach some patients), there is justification for an RCT with a waiver of informed consent.

Because the RCT will only change how the intervention is allocated (from operational convenience to randomization), it will not require informed consent. This approach was used by Dr. Pevnick in his recent RCT.

We are asking for a waiver of informed consent for the following reasons:

1. Feasibility: Our primary aim in this study is to examine utilization. Our study design is powered to detect a clinically meaningful difference between the two study groups (intervention and control), but to detect this difference, we require a very large sample size. We are looking to recruit several thousand patients in the study period. Given the high workload of the clinical pharmacists participating in this study, it would not be feasible for us to conduct this study without a waiver of consent [See Weijer C, Grimshaw JM, Taljaard M, Binik A, Boruch R, Brehaut JC, et al. Ethical issues posed by cluster randomized trials in health research. Trials. 2011;12:100]
2. Minimal Risk: The risks to the patients are minimal. After the medication history is taken for patients, the patients are randomized into either the treatment or control group. However, if a clinician believes that the patient would benefit from the pharmacist consult and/or post-discharge phone call, the patient will meet with the pharmacist regardless of whether they are in the study. Thus, any patient who needs the pharmacist services will receive the appropriate care. The pharmacist participating in the intervention will provide a consult to the physician about medications that should be changed, but the physician will have the final say about any medication changes. This is equivalent to the physician consulting an online resource or receiving a computerized alert about medication recommendations (clinical decision support). Participating in the study will not adversely affect the rights or welfare of the participants since patients who request a meeting with a clinical pharmacist or situations where the attending physician believes the patient should meet with the clinical pharmacist will have every right to do so.
3. Reducing Risk: Since this study is a very low risk intervention, we believe that adding a signed consent form would actually introduce more risk because it would now introduce a document that could identify the participants. We are planning on analyzing the study data at the completion of the intervention and the data will be extracted and only accessed by select study staff. Incorporating documents with PHI may increase the risk for patients of loss of confidentiality.
4. Decrease of Data Validity and Quality: Patients who are randomized to the control group may be more likely not to participate in the study (a phenomenon known as “resentful demoralization”) so there may be systematic bias introduced if patients are asked to consent, which could decrease the validity of study results [see Rebers S, Aaronson NK, Van Leeuwen FE, Schmidt MK. Exceptions to the rule of informed consent for research with an intervention. BMC medical ethics. 2016 Dec;17(1):9.].

The risk to subjects is no more than minimal risk to privacy. We have put several measures in place to protect the privacy of subjects and confidentiality of the data:

[1] Personally identifying information will only be available to study personnel.

[2] All identifiable (and de-identified) data will be in electronic format. These data will be stored and used entirely within CSMC computer systems on the CSMC network, where appropriate technical safeguards (including authentication, security and virus protection) are in place. Since the data originates on this network, the increase in risk is minimal.

[3] All identifiers will be destroyed at the end of the study (the research data itself will be kept for seven years after completion of the research).

During training, trainers will emphasize to pharmacists participating in this study additional, clinically-indicated services may be provided to control group patients to ensure that it is clear that routine services that are part of the PHARM-DC and that the toolkit should not be withheld from the control group if they are clinically indicated. This will ensure that patients in the control group will receive appropriate services if needed.

Focus Groups and Interviews

For the focus groups, we will provide an information sheet outlining the purpose, risks, and benefits of the study intervention. A member of the study staff will go over the information sheet.

Information sheets will be Institutional Review Board (IRB)-approved, and the participant will be asked to read and review the document. The investigator will explain the research study to the participant and answer any questions that may arise. A verbal explanation will be provided in terms suited to the participant's comprehension of the purposes, procedures, and potential risks of the study and of their rights as research participants. Participants will have the opportunity to carefully review the written information sheet and ask questions prior to participating in the focus group. Participants will be informed that participation is voluntary and that they may withdraw from the study at any time, without prejudice.

Focus groups and interviews present no more than minimal risk. Signed consent will not be obtained for focus group/interview participants. The focus groups/interviews will be conducted in areas which the staff frequently occupy as part of their daily tasks and the questions will require no more disclosure of information than would be routinely occur in their conventional work environment. Attendance and participation in the focus group will be considered consent to participate. It is also possible that interviews and/or focus groups will take place via video conferencing software (e.g. Webex) or over the phone.

The qualitative information will not include any sensitive or incriminating information. Our study team will not collect more data than is necessary to answer the research question. However, questions could elicit negative comments about their health system. Focus group sessions and interviews will be recorded via audiotape and transcribed by research team members or a professional transcribing service. The audiotapes will be stored in locked file cabinets within locked offices, and electronic transcriptions will also be housed on firewall protected servers, will not include subject names, and may only be accessed by select members of the research team. Names will be stripped from the transcribed interviews and focus group responses will reported only in aggregate. Names will be removed from the documentation material use for data analyses.

The observations will take place at the two study sites (CSMC and BWH). They may include observations of patients/caregiver interactions. Researchers will not collect any PHI or identifiable information while observing these interactions. The qualitative information obtained during observations will not include any sensitive or incriminating information, or any information that could impact a subject's employability. All observation data will be recorded with no identifiable information. Subjects will be provided with the information sheet which includes information on the observation component of the study and will be verbally notified that observations will occur prior to observations commencing.

Focus group and interview participants will not sign a consent form. Keeping signed consent forms will only increase the chance of a breach of confidentiality. Further, no identifying information, including any PHI elements, will be asked during the focus group. If a subject mentions any PHI elements during the focus group, it will not be transcribed and will be deleted from the audio-recording.

Time and Motion Study

Eligible participants (pharmacists working on the intervention) will be approached at the beginning of the day and will be given an information sheet. The information sheet will include information about the participant observation. The investigator will explain the research study to the participant and answer any questions that may arise. A verbal explanation will be provided in terms suited to the participant's comprehension of the purposes, procedures, and potential risks of the study and of their rights as research participants.

9.1.2 CONFIDENTIALITY AND PRIVACY

Participant confidentiality and privacy is strictly held in trust by the participating investigators, their staff, and the sponsor(s) and their interventions. Therefore, the study protocol, documentation, data, and all other information generated will be held in strict confidence. No information concerning the study or the data will be released to any unauthorized third party without prior written approval of the sponsor.

All research activities will be conducted in as private a setting as possible.

The study monitor, other authorized representatives of the sponsor, representatives of the Institutional Review Board (IRB), regulatory agencies or pharmaceutical company supplying study product may inspect all documents and records required to be maintained by the investigator, including but not limited to, medical records (office, clinic, or hospital) and pharmacy records for the participants in this study. The clinical study site will permit access to such records.

The study participant's contact information will be securely stored at each clinical site for internal use during the study. At the end of the study, all records will continue to be kept in a secure location for as long a period as dictated by the reviewing IRB, Institutional policies, or sponsor requirements.

Study participant research data, which is for purposes of statistical analysis and scientific reporting, will be transmitted to and stored at CSMC. This will not include the participant's contact or identifying information. Rather, individual participants and their research data will be identified by a unique study identification number. The study data entry and study management systems used by clinical sites and by CSMC research staff will be secured and password protected. At the end of the study, all study databases will be de-identified and archived at the CSMC.

Qualitative Study

The qualitative data will be stripped of identifiers and will be analyzed by researchers at the University of Iowa. The qualitative information will not include any sensitive or incriminating

information. Our study team will not collect more data than is necessary to answer the research question. However, questions could elicit negative comments about their health system. Focus group sessions and interviews will be recorded via audiotape and transcribed by research team members or a professional transcribing service. The audiotapes will be stored in locked file cabinets within locked offices, and electronic transcriptions will also be housed on firewall protected servers, will not include subject names, and may only be accessed by select members of the research team. Names will be stripped from the transcribed interviews and focus group responses will reported only in aggregate. Names will be removed from the documentation material use for data analyses.

We have added relevant text to the information sheets to note that investigators may conduct observations with pharmacists. Participants will be informed that participation is voluntary and that they may withdraw from the study at any time, without prejudice. The qualitative information will not include any sensitive or incriminating information. Our study team will not collect more data than is necessary to answer the research question. However, questions could elicit negative comments about their health system. Observations will be given codes and they will be stored separately with a linking list. No patient-level data will be recorded.

Time-and-Motion Study

Patients will be assigned a unique identifier within the online data collection tool so that no PHI is transmitted over the internet. Only IRB-approved study staff will have access to the linking file which will be stored separately from patient-level data on Box. Data will only be accessed on a password protected, encrypted study site computer.

Pharmacists will be provided a study information sheet prior to observations taking place, and patients who may have interactions with the pharmacists will be told that this is part of an ongoing study examining pharmacist activities. Participants will be reminded at the beginning of the observation that they are not required to participate in the observations and can stop the observation at any time.

9.1.3 KEY ROLES AND STUDY GOVERNANCE

Provide the name and contact information of the Principal Investigator and the Medical Monitor.

**Principal Investigator**
Joshua Pevnick, MD, MS
CSMC
8700 Beverly Blvd, West Hollywood, CA 90048
310-423-6976
Joshau.pevnick@cshs.org

9.1.4 SAFETY OVERSIGHT

Safety oversight will be under the direction of a Data and Safety Monitoring Board (DSMB) composed of individuals with the appropriate expertise. Members of the DSMB are independent from the study conduct and free of conflict of interest, or measures should be in place to minimize perceived conflict of interest. The DSMB will meet at least semiannually to review

adverse events. The DMSB will operate under the rules of an approved charter that will be written and reviewed at the organizational meeting of the DSMB. At this time, each data element that the DSMB needs to assess will be clearly defined. The DSMB will provide its input to NIA.

9.1.5 DATA HANDLING AND RECORD KEEPING

Each participating site will maintain appropriate medical and research records for this trial, in compliance with ICH GCP and regulatory and institutional requirements for the protection of confidentiality of participants. As part of participating in a NIH-sponsored or NIH-affiliated study, each site will permit authorized representatives of the NIH, sponsor, and regulatory agencies to examine (and when permitted by applicable law, to copy) clinical records for the purposes of quality assurance reviews, audits, and evaluation of the study safety, progress, and data validity. Describe in this section who will have access to records.

9.1.5.1 DATA COLLECTION AND MANAGEMENT RESPONSIBILITIES

Clinical data (including adverse events (AEs), concomitant medications, and expected adverse reactions data) and clinical laboratory data will be entered into RED-Cap, a 21 CFR Part 11-compliant data capture system provided by CSMC. The data system includes password protection and internal quality checks, such as automatic range checks, to identify data that appear inconsistent, incomplete, or inaccurate. Clinical data will be entered directly from the source documents.

To evaluate trial outcomes, data from the Brigham and Women's Hospital site will be transmitted to the coordinating study team at Cedars-Sinai. Data transmission, storage, and management will adhere to the following procedures for data security and privacy:

- **Secure storage**: Data will be housed in a password-protected, HIPAA-compliant secure storage system, Box, within the Cedars-Sinai network with access restricted to approved members of the research team.
- **Limited Access**: Private identifiable information, will be accessible only to IRB approved study team members with current IRB training.
- **Unique ID Numbers**: Each patient will be assigned a unique ID number.
- **Removal of Identifiers**: Direct identifiers (like name or MRN) will be removed from the research records and destroyed as soon as scientifically possible and maintained only as long as necessary to abstract, analyze and verify data.
- **Storage of Physical Records**: This study will not include the storage of physical records.

9.1.5.2 STUDY RECORDS RETENTION

Study documents should be retained for a minimum of 2 years after the last approval of a marketing application in an International Conference on Harminosation (ICH) region and until there are no pending or contemplated marketing applications in an ICH region or until at least 2 years have elapsed since the formal discontinuation of clinical development of the study intervention. These documents should be retained for a longer period, however, if required by local regulations. No records will be destroyed without the written consent of the sponsor, if applicable.

9.1.6 PUBLICATION AND DATA SHARING POLICY

This study will be conducted in accordance with the following publication and data sharing policies and regulations:

National Institutes of Health (NIH) Public Access Policy, which ensures that the public has access to the published results of NIH funded research. It requires scientists to submit final peer-reviewed journal manuscripts that arise from NIH funds to the digital archive PubMed Central upon acceptance for publication.

This study will comply with the NIH Data Sharing Policy and Policy on the Dissemination of NIH-Funded Clinical Trial Information and the Clinical Trials Registration and Results Information Submission rule. As such, this trial will be registered at ClinicalTrials.gov, and results information from this trial will be submitted to ClinicalTrials.gov. In addition, every attempt will be made to publish results in peer-reviewed journals. Data from this study may be requested from other researchers 2 years after the completion of the primary endpoint by contacting Josh Pevnick, the study PI.

9.1.7 CONFLICT OF INTEREST POLICY

The independence of this study from any actual or perceived influence, such as by the pharmaceutical industry, is critical. Therefore, any actual conflict of interest of persons who have a role in the design, conduct, analysis, publication, or any aspect of this trial will be disclosed and managed. Furthermore, persons who have a perceived conflict of interest will be required to have such conflicts managed in a way that is appropriate to their participation in the design and conduct of this trial. The study leadership in conjunction with the <specify NIH Institute or Center (IC)> has established policies and procedures for all study group members to disclose all conflicts of interest and will establish a mechanism for the management of all reported dualities of interest.

9.2 PROTOCOL AMENDMENT HISTORY

Version	Date	Description of Change	Brief Rationale
4.0	10/22/2020	Changed inclusion criteria of age from "≥65 years old" to "≥55 years old".	After 43 weeks of randomization, the minimum patient enrollment age was reduced from 65 years to 55 years to enhance study enrollment due to unexpected COVID-19 impacts on participant enrollment.
5.0	12/15/2020	1. Added exclusion criteria for patients with suspected or confirmed diagnosis of COVID-19.<br><br>2. Added description of pharmacist communication with key inpatient providers as part of the intervention.	1. Patients admitted with a suspected or confirmed diagnosis of COVID-19 constitute a significantly different patient population than that which is current under study in the PHARM-DC trial.<br><br>2. This description was added to more clearly describe activities being delivered as part of the intervention by pharmacists at both study sites.
6.0	2/16/2021	Added sections:<br><br>8.4.5. Examine effects of PHARM-DC intervention on initiation and deprescribing of benzodiazepine-receptor agonists<br><br>8.4.6 Examine effects of PHARM-DC intervention on fall-related injuries among older adults with Alzheimer's disease and related dementias	These sections were added to describe pre-planned secondary data analyses.
7.0	4/20/2021	Updating the definition of adverse events/severe adverse events in Section 7.2.4	To clarify the time period and frequency for event assessment and follow-up
8.0	6/25/2021	Updates to time-and-motion study procedures	To clarify data collection, data management, and data analyses for the time-and-motion study
9.0	2/16/2022	1. Clarified handling of elective readmissions in calculating the primary outcome of 30-day post-	To clarify study procedures, additional secondary subgroup analyses, and

		discharge utilization<br><br>2. Clarified procedures for study coordinators to stop randomizing additional patients if pharmacist workload capacity is exceeded<br><br>3. Updated 1 reference that was recently published<br><br>4. Added secondary subgroup analyses, including outcome comparisons by:<br>(a) patient age group,<br>(b) limited English proficiency as a proxy for low medication literacy,<br>(c) before and after historical changes to the intervention and control arm,<br>(d) restriction to medication-related post-discharge utilization, and<br>(e) restriction to 7-day post-discharge utilization.	update references.
10.0	3/17/2022	1. Notification of request for a "one-time operational report" without switching our data source. For quality assurance, one-time operational report(s) will be obtained from the Emergency Department and cross-checked with the EIS report to ensure all patients who may be eligible for the study are being accurately identified. We believe our existing EIS reporting/code may contain errors and need to obtain the operational report in order to ensure validity of our subject recruitment.<br><br>2. Specified demographic variables to be collected from participating pharmacists. Subjects (pharmacists) were already previously informed of demographic data collection. Information will be collected via the pharmacist demographic data collection form (attached)	1. Operational report needed form the ED to verify EIS daily reports are sufficiently capturing all potential, eligible patients for PHARM-DC enrollment.<br><br>2. Confirmation and approval of specific pharmacist demographic variables requested for time and motion manuscript.<br><br>3. IRB analyst instructed this clerical change when requesting information for the operational report clarification.

		forwarded to their department manager.<br><br>3. Clarified that the daily admissions reports used for subject enrollment are and have been generated by EIS.	
11.0	6/2/2022	1. Added patient identifiers to be obtained for Dr. Gotanda's secondary analysis of trial data<br><br>2. Added secondary outcomes: 30-day mortality and effect of the intervention on each type of utilization considered separately (ED visits alone, observations stays alone, readmissions alone)<br><br>3. Moved description (list) of pharmacist demographics from secondary outcomes to Study Design section.	To link Medicare claims data to trial data<br><br>Specification of additional secondary outcomes to be assessed for main trial.<br><br>Moved to correct location as these were not secondary outcomes.
12.0	9/9/2022	Clarified recruitment strategies for focus groups and interviews under Section 5.3	Clarified that an honorarium will be offered to nurses and physicians as a strategy to enhance recruitment of these professionals for focus groups and/or interviews.
13.0	11/9/2022	Clarified data collection and management procedures for Dr. Gotanda's secondary analysis under sections 8.4.6 and 9.1.5.1.	Clarified procedures for maintaining privacy and security of patient identifiers transmitted from the Brigham and Women's Hospital site to the coordinating study team at Cedars-Sinai.
14.0	11/17/2022	Increased focus group honorariums by 25% for physicians (from $200 to $$250) and for nurses (from $100 to $125).	Requesting increase to focus group honorariums by 25% to account for gift cards being taxed as taxable income for the focus group participants who are employees of the study site.
15.0	12/29/2022	Added Appendix A	To clarify data elements to be extracted from the electronic health records at study sites for the purpose of evaluating trial outcomes
16.0	8/25/2023	Added Section 8.4.7 (secondary analysis)	To clarify secondary analysis for propensity score

			matching at BWH

10 APPENDIX A

List of data elements to be requested from electronic health records at study sites

Unless otherwise specified, the following data elements are requested at the time of admission for the index hospital admission (CSN's will be provided):

1. Index hospital admission date
2. Index hospital discharge date
3. Index admission encounter type: emergency department, inpatient, or observation visit
4. Body mass index (BMI)
5. Charlson Comorbidity Index (CCI) and/or Elixhauser Comorbidity Index
    a. Age-adjusted CCI
6. Count of the number of hospital admissions in 3 years preceding index admission
7. Count of medications prior to admission
8. Count of medications at discharge
9. Date of birth
10. Date of death
11. Discharge location
12. Education level
13. Estimated glomerular filtration rate (mL/min)
14. Ethnicity
15. ICD-9 and ICD-10 diagnosis codes (from physician billing, hospital billing, and problem list) at admission
16. ICD-9 and ICD-10 diagnosis codes (from physician billing, hospital billing, and problem list) at discharge
17. Date of diagnosis (associated with diagnosis codes, see #15 and #16)
18. Insurance type (e.g., Medicare, Medicaid, dual eligible, private, uninsured)
19. Interpreter needed (yes/no)?
20. Length of stay
21. Marital status
22. Medication Adherence and Literacy Scale (MedAL) score at hospital discharge
23. Medication names prior to admission
    a. Medication sigs, medication meta-data (class, subclass, dose dispensed etc.), if available
24. Medication names at discharge
25. Medication category (i.e., anticoagulant, antiplatelet, antihypoglycemics)
26. Patient's home address
    a. National Deprivation Index
27. Primary language
28. Race
29. Sex/gender

30. Social history (including smoking status, alcohol use status, and employment status), if available
31. Zip code

The following data elements are requested for all encounters within 30 days after hospital discharge of the index admission:

32. CSN's for all encounters within 30 days after hospital discharge
33. Encounter dates
34. Encounter type (e.g., emergency department, inpatient, or observation visit)
35. Location of encounter (e.g., hospital, emergency department, office)
36. Was the encounter preceded by an emergency department visit (yes/no)?
37. For encounters, all available ICD-9 and ICD-10 diagnosis codes at admission
38. For encounters, all available ICD-9 and ICD-10 diagnosis codes at discharge
39. For encounters, all available procedure codes (ICD, CPT, Healthcare Common Procedure Coding System [HCPCS] codes) during encounter
40. For encounters, all available external cause of injury codes ("E-codes") at admission
41. For encounters, all available external cause of injury codes ("E-codes") at discharge
42. For encounters, date of diagnosis, procedure, and external cause of injury (see codes for #37-#41)
43. Source of encounter data for #37-41 (e.g., institutional claims, professional claims), if available

11 REFERENCES

1. California Office of Statewide Health Planning and Development. Cedars-Sinai Medical Center: Hospital Facility Details & References. *http://gis.oshpd.ca.gov/atlas/places/facility/106190555*. Published 2013. Accessed May 19, 2016, 2016.

2. U.S. Bureau of Labor Statistics. Overview of BLS Wage Data by Area and Occupation. *https://www.bls.gov/bls/blswage.htm*. Published 2017. Updated February 28, 2017. Accessed March 13, 2019, 2019.

3. Long JS, Freese J. *Regression models for categorical dependent variables using Stata.* Stata press; 2006.

4. Imamura T, Hirono N, Hashimoto M, et al. Fall-related injuries in dementia with Lewy bodies (DLB) and Alzheimer's disease. *Eur J Neurol.* 2000;7(1):77-79.

5. Centers fo Medicare and Medicaid Services. Chronic Conditions Data Warehouse Condition Categories. *https://www2.ccwdata.org/web/guest/condition-categories*. Published 2019. Accessed January 28, 2020.

---

[i] Tsilimingras D, Schnipper J, Duke A, Agens J, Quintero S, Bellamy G, Janisse J, Helmkamp L, Bates DW. Post-Discharge Adverse Events Among Urban and Rural Patients of an Urban Community Hospital: A Prospective Cohort Study. Journal of general internal medicine. 2015;30(8):1164-71. Epub 2015/03/31. doi: 10.1007/s11606-015-3260-3. PubMed PMID: 25822112; PMCID: Pmc4510218.

[ii] Kripalani S, Roumie CL, Dalal AK, Cawthon C, Businger A, Eden SK, Shintani A, Sponsler KC, Harris LJ, Theobald C, Huang RL, Scheurer D, Hunt S, Jacobson TA, Rask KJ, Vaccarino V, Gandhi TK, Bates DW, Williams MV, Schnipper JL. Effect of a pharmacist intervention on clinically important medication errors after hospital discharge: a randomized trial. Annals of internal medicine. 2012;157(1):1-10. Epub 2012/07/04. doi: 10.7326/0003-4819-157-1-201207030-00003. PubMed PMID: 22751755; PMCID: Pmc3575734.

[iii] Forster AJ, Murff HJ, Peterson JF, Gandhi TK, Bates DW. The incidence and severity of adverse events affecting patients after discharge from the hospital. Annals of internal medicine. 2003;138(3):161-7. Epub 2003/02/01. PubMed PMID: 12558354.

[iv] Burns JM, Sneddon I, Lovell M, McLean A, Martin BJ. Elderly patients and their medication: a post-discharge follow-up study. Age and ageing. 1992;21(3):178-81. Epub 1992/05/01. PubMed PMID: 1615779.

[v] Ernst FR, Grizzle AJ. Drug-related morbidity and mortality: updating the cost-of-illness model. Journal of the American Pharmaceutical Association (Washington,DC : 1996). 2001;41(2):192-9. Epub 2001/04/12. PubMed PMID: 11297331.

[vi] Schnipper JL, Kirwin JL, Cotugno MC, et al. Role of pharmacist counseling in preventing adverse drug events after hospitalization. Archives of Internal Medicine. 2006;166(5):565-71. doi: 10.1001/archinte.166.5.565.

[vii] Cresswell KM, Fernando B, Mckinstry B, Sheikh A. Adverse drug events in the elderly. Br Med Bull. 2007;83:259-74

[viii] Mansur N, Weiss A, Beloosesky Y. Relationship of In-Hospital Medication Modifications of Elderly Patients to Postdischarge Medications, Adherence, and Mortality. Annals of Pharmacotherapy. 2008;42(6):783-9. doi: 10.1345/aph.1L070.

[ix] Elliott RA, O'Callaghan CJ. Impact of Hospitalisation on the Complexity of Older Patients' Medication Regimens and Potential for Regimen Simplification. Journal of Pharmacy Practice and Research. 2011;41(1):21-5. doi: 10.1002/j.2055-2335.2011.tb00060.x.

[x] Frankenthal D, Lerman Y, Lerman Y. The impact of hospitalization on potentially inappropriate prescribing in an acute medical geriatric division. International Journal of Clinical Pharmacy. 2014;37(1):60-7. doi: 10.1007/s11096-014-0040-9.

[xi] Beers MH, Sliwkowski J, Brooks J. Compliance with medication orders among the elderly after hospital discharge. Hosp Formul 1992;27:720–724.

[xii] Kanaan AO, Donovan JL, Duchin NP, Field TS, Tjia J, Cutrona SL, Gagne SJ, Garber L, Preusse P, Harrold LR, Gurwitz JH. Adverse Drug Events After Hospital Discharge in Older Adults: Types, Severity, and Involvement of Beers Criteria Medications. Journal of the American Geriatrics Society. 2013;61(11):1894-9. doi: 10.1111/jgs.12504.

[xiii] Teymoorian SS, Dutcher D, Woods M. Association between postdischarge adverse drug reactions and 30-day hospital readmission in patients aged 80 and older. J Am Geriatr Soc. 2011;59(5):948-9. Epub 2011/05/17. doi: 10.1111/j.1532-5415.2011.03376.x. PubMed PMID: 21568972.

[xiv] Bonnet-Zamponi D, d'Arailh L, Konrat C, Delpierre S, Lieberherr D, Lemaire A, Tubach F, Lacaille S, Legrain S. Drug-related readmissions to medical units of older adults discharged from acute geriatric units: results of the Optimization of Medication in AGEd multicenter randomized controlled trial. J Am Geriatr Soc. 2013;61(1):113-21. Epub 2012/12/21. doi: 10.1111/jgs.12037. PubMed PMID: 23252914.

[xv] Feigenbaum P, Neuwirth E, Trowbridge L, Teplitsky S, Barnes CA, Fireman E, Dorman J, Bellows J. Factors contributing to all-cause 30-day readmissions: a structured case series across 18 hospitals. Med Care. 2012;50(7):599-605. Epub 2012/02/23. doi: 10.1097/MLR.0b013e318249ce72. PubMed PMID: 22354212.

[xvi] Forster AJ, Murff HJ, Peterson JF, Gandhi TK, Bates DW. The incidence and severity of adverse events affecting patients after discharge from the hospital. Ann Intern Med. 2003; 138(3):161- 7.

[xvii] Forster AJ, Murff HJ, Peterson JF, Gandhi TK, Bates DW. Adverse drug events occurring following hospital discharge. Journal of general internal medicine. 2005;20(4):317-23. Epub 2005/04/29. doi: 10.1111/j.1525-1497.2005.30390.x. PubMed PMID: 15857487; PMCID: Pmc1490089.

[xviii] Bell DS, Cretin S, Marken RS, Landman AB. A Conceptual Framework for Evaluating Outpatient Electronic Prescribing Systems Based on Their Functional Capabilities. Journal of the American Medical Informatics Association : JAMIA. 2004;11(1):60-70. doi: 10.1197/jamia.M1374. PubMed PMID: PMC305459.

[xix] McKibbon KA, Lokker C, Handler SM, Dolovich LR, Holbrook AM, O'Reilly D, Tamblyn R, Hemens BJ, Basu R, Troyan S, Roshanov PS, Archer NP, Raina P. Enabling Medication Management Through Health Information Technology. Evidence Report/Technology Assessment No. 201. (Prepared by the McMaster University Evidence-based Practice Center under Contract HHSA 290-2007-10060-I). AHRQ Publication No. 11-E008-EF. Rockville MD: Agency for Healthcare Research and Quality. April 2011

[xx] Kuijpers MAJ, van Marum RJ, Egberts ACG, Jansen PAF. Relationship between polypharmacy and underprescribing. British Journal of Clinical Pharmacology. 2008;65(1):130-3. doi: 10.1111/j.1365-2125.2007.02961.x. PubMed PMID: PMC2291281.

[xxi] Kripalani S, Roumie CL, Dalal AK, Cawthon C, Businger A, Eden SK, Shintani A, Sponsler KC, Harris LJ, Theobald C, Huang RL, Scheurer D, Hunt S, Jacobson TA, Rask KJ, Vaccarino V, Gandhi TK, Bates DW, Williams MV, Schnipper JL. Effect of a pharmacist intervention on clinically important medication errors after hospital discharge: a randomized trial. Annals of internal medicine. 2012;157(1):1-10. Epub 2012/07/04. doi: 10.7326/0003-4819-157-1-201207030-00003. PubMed PMID: 22751755; PMCID: PMC3575734.

[xxii] Bell SP, Schnipper JL, Goggins K, Bian A, Shintani A, Roumie CL, Dalal AK, Jacobson TA, Rask KJ, Vaccarino V, Gandhi TK, Labonville SA, Johnson D, Neal EB, Kripalani S. Effect of Pharmacist Counseling Intervention on Health Care Utilization Following Hospital Discharge: A Randomized Control Trial. Journal of general internal medicine. 2016;31(5):470-7. Epub

2016/02/18. doi: 10.1007/s11606-016-3596-3. PubMed PMID: 26883526; PMCID: PMC4835388.

[xxiii] Budnitz DS, Lovegrove MC, Shehab N, Richards CL. Emergency Hospitalizations for Adverse Drug Events in Older Americans. New England Journal of Medicine. 2011;365(21):2002-12. doi: 10.1056/NEJMsa1103053. PubMed PMID: 22111719.

[xxiv] Pippins JR, Gandhi TK, Hamann C, Ndumele CD, Labonville SA, Diedrichsen EK, Carty MG, Karson AS, Bhan I, Coley CM, Liang CL, Turchin A, McCarthy PC, Schnipper JL. Classifying and predicting errors of inpatient medication reconciliation. Journal of general internal medicine. 2008;23(9):1414-22. Epub 2008/06/20. doi: 10.1007/s11606-008-0687-9. PubMed PMID: 18563493; PMCID: Pmc2518028.

[xxv] Pevnick JM, Nguyen C, Jackevicius CA, Palmer KA, Shane R, Cook-Wiens G, Rogatko A, Bear M, Rosen O, Seki D, Doyle B, Desai A, Bell DS. Improving admission medication reconciliation with pharmacists or pharmacy technicians in the emergency department: A randomised controlled trial. BMJ Qual Saf. 2018 Jul;27(7):512-520. doi: 10.1136/bmjqs-2017-006761. Epub 2017 Oct 6. PMID: 28986515; PMCID: PMC5912995.

[xxvi] Maust DT, Gerlach LB, Gibson A, Kales HC, Blow FC, Olfson M. Trends in Central Nervous System-Active Polypharmacy Among Older Adults Seen in Outpatient Care in the United States. JAMA internal medicine. 2017.

[xxvii] Bear, M Impact of pharmacist post-discharge phone calls on hospital readmissions and patient medication adherence [Abstract]. Presented at Western States Conference for Pharmacy Residents, Fellows, and Preceptors, May, 2014.

[xxviii] Bero LA, Grilli R, Grimshaw JM, Harvey E, Oxman AD, Thomson MA. Closing the gap between research and practice: an overview of systematic reviews of interventions to promote the implementation of research findings. BMJ: British Medical Journal. 1998;317(7156):465.

[xxix] Shea BJ, Grimshaw JM, Wells GA, Boers M, Andersson N, Hamel C, Porter AC, Tugwell P, Moher D, Bouter LM. Development of AMSTAR: a measurement tool to assess the methodological quality of systematic reviews. BMC Medical Research Methodology. 2007;7(1):10. doi: 10.1186/1471-2288-7-10.

[xxx] Nasir K, Lin Z, Bueno H, Normand S-LT, Drye EE, Keenan PS, Krumholz HM. Is Same-Hospital Readmission Rate a Good Surrogate for All-Hospital Readmission Rate? Medical Care. 2010;48(5):477-81. doi: 10.1097/MLR.0b013e3181d5fb24. PubMed PMID: 00005650-201005000-00013.

[xxxi] http://www.chiamass.gov

[xxxii] Strauss AL. Qualitative Analysis for Social Scientists. Cambridge: Cambridge University Press; 1987

[xxxiii] Hill CE, Knox S, Thompson BJ, Williams EN, Hess SA, Ladany N. (2005). *Consensual qualitative research: An update.* Journal of Counseling Psychology, 52, 196–205

[xxxiv] Bazeley P. Computerized data analysis for mixed methods research. In: Tashakkori A, Teddlie C, eds. *Mixed methodology: combining qualitative and quantitative approaches*. Thousand Oaks, CA: Sage; 2003.
[xxxv] Frick KD. Micro-Costing Quantity Data Collection Methods. Medical care. 2009;47(7 Suppl 1):S76-S81.
[xxxvi] Poon EG, Keohane C, Featherstone E, et al. Impact of barcode medication administration technology on how nurses spend their time on clinical care. AMIA Annu Symp Proc 2006:1065.
[xxxvii] Keohane CA, Bane AD, Featherstone E, et al. Quantifying nursing workflow in medication administration. J Nurs Adm 2008;38(1):19-26.
[xxxviii] Ungar JP, Gandhi TK, Poon EG, et al. Impact of ambulatory computerized physician order entry on clinicians' time. AMIA Annu Symp Proc 2008:1158.
[xxxix] Huang KT, Minahan J, Brita-Rossi P, et al. All Together Now: Impact of a Regionalization and Bedside Rounding Initiative on the Efficiency and Inclusiveness of Clinical Rounds. J Hosp Med 2017;12(3):150-56.
[xl] Nguyen CB, Shane R, Bell DS, Cook-Wiens G, Pevnick JM. A Time and Motion Study of Pharmacists and Pharmacy Technicians Obtaining Admission Medication Histories. J Hosp Med. 2017;12(3):180-3. Epub 2017/03/09. doi: 10.12788/jhm.2702. PubMed PMID: 28272596.
