# Supplementary material for: Pharmacist-Led Discharge Care to Reduce Postdischarge Health Care Utilization: A Randomized Clinical Trial
Source: JAMA Netw Open. 2026 Mar 17;9(3):e260719. doi: 10.1001/jamanetworkopen.2026.0719 (PMC13446754; doi:10.1001/jamanetworkopen.2026.0719)
Supplement: Supplementary file 2 [file jamanetwopen-e260719-s002.pdf]

Supplementary Online Content

Pevnick JM, Kennelty K, Nguyen AT, et al; PHARM-DC Group. Pharmacist-led discharge care to reduce postdischarge health care utilization: a randomized clinical trial. *JAMA Netw Open*. 2026;9(3):e260719. doi:10.1001/jamanetworkopen.2026.0719

eFigure. Patient Flow

eTable 1. Utilization Outcomes Evaluated Separately and Postdischarge Mortality

eTable 2. Same-Hospital 30-Day Unplanned Utilization Stratified by Medication Adherence and Literacy (MedAL) Score

eTable 3. Tests for Effect Modification

eTable 4. Effect of Intervention on Postdischarge Utilization Among Patients With Low Medication Adherence and Literacy

eReference

This supplementary material has been provided by the authors to give readers additional information about their work.

© 2026 Pevnick JM et al. *JAMA Network Open*.

eFigure. Patient flow

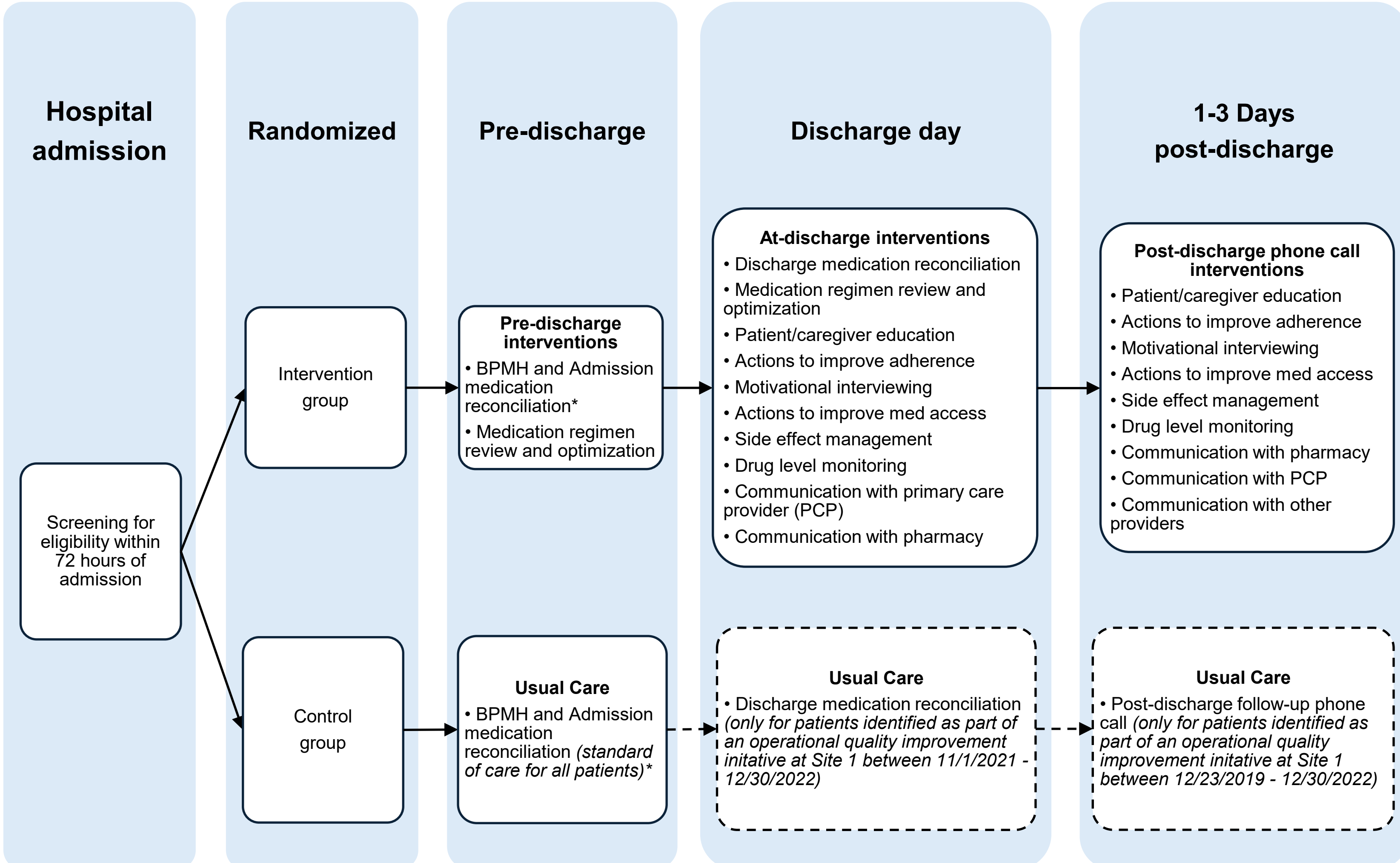

Notes. Dashed arrows and dashed boxes represent variations in usual care. Study enrollment period was from 12/23/2019 through 12/30/2022. Besides managing patient caseloads, other pharmacist work duties included: supervising pharmacy technicians, precepting pharmacy students/residents, and clinical documentation.

*Admission medication reconciliation occurred after the decision for hospital admission, including in some cases prior to screening, enrollment, and randomization.
BPMH: best possible medication history.

© 2026 Pevnick JM et al. *JAMA Network Open*.

eTable 1. Utilization Outcomes Evaluated Separately and Postdischarge Mortality

**Outcome**	**Usual Care**	**Intervention**	**Absolute Reduction (95% CI)**	**p-value (Chi-square)**
Same-Hospital 30-Day Unplanned ED Utilization (n=6238)	206/3112 (6.6%)	165/3126 (5.3%)	1.3% (0.2, +2.5)	0.03**
Same-Hospital 30-Day Unplanned Observation Stays (n=6238)	51/3112 (1.6%)	60/3126 (1.9%)	-0.3% (-0.9, +0.4)	0.40
Same-Hospital 30-Day Unplanned Readmissions (n=6238)	534/3112 (17.2%)	541/3126 (17.3%)	-0.1% (-2.0, +1.7)	0.88
All-Hospital 30-Day ED Utilization (n=4472)*	556/2242 (24.8%)	514/2230 (23.0%)	1.8% (-0.8, +4.3)	0.17
All-Hospital 30-Day Observation Stays (n=4472)*	183/2242 (8.2%)	194/2230 (8.7%)	-0.5% (-2.2, +1.1)	0.52
All-Hospital 30-Day Readmissions (n=4472)*	430/2242 (19.2%)	424/2230 (19.0%)	0.2% (-2.1, +2.5)	0.89
30-Day Mortality	102/3112 (3.3%)	103/3126 (3.3%)	0.0% (-0.9, +0.9)	0.97

*n=1766 (870 usual care, 896 intervention) excluded due to not having fee-for-service Medicare to allow assessment of utilization at non-study site hospitals via CMS claims

** did not meet the statistical significance threshold of 0.014 determined by using the Benjamini-Hochberg procedure to account for testing seven hypotheses, using a false discovery rate of 0.1

© 2026 Pevnick JM et al. *JAMA Network Open*.

eTable 2. Same-Hospital 30-Day Unplanned Utilization Stratified by Medication Adherence and Literacy (MedAL) Score

Medication Adherence and Literacy	Usual Care	Intervention	Absolute Reduction (95% CI)	Stratified p-value*
Low	69/240 (28.8%)	64/349 (18.3%)	10.4% (+3.4%, +17.4%)	0.003
Moderate	105/582 (18%)	131/737 (17.8%)	0.3% (-3.9%, +4.4%)	0.90
High	178/958 (18.6%)	198/1108 (17.9%)	0.7% (-2.6%, +4.1%)	0.68
Not documented	150/834 (18%)	85/442 (19.2%)	-1.3% (-5.8%, +3.3%)	0.59
No intent to assess with MedAL**	104/498 (20.9%)	101/490 (20.6%)	0.3% (-4.8%, +5.3%)	0.92

*Z-test for proportions

**Deemed to be likely missing at random because enrollment occurred after MedAL score assessment stopped being used at the enrolling site

© 2026 Pevnick JM et al. *JAMA Network Open*.

eTable 3. **Tests for Effect Modification.** Benjamini-Hochberg Procedure to Account for Multiple Testing

Subgroup	p-value for interaction	Rank	Critical value (i/m) * Q	p-value smaller than critical value?
Medication adherence and literacy	0.0117	1	0.0125	Yes
Median income by census tract	0.2802	2	0.0250	No
≥10 chronic preadmission prescriptions	0.3784	3	0.0375	No
Age	0.7703	4	0.0500	No
Heart failure	0.7921	5	0.0625	No
Sex	0.8233	6	0.0750	No
≥3 high-risk chronic preadmission prescriptions	0.8271	7	0.0875	No
Study site	0.9987	8	0.1000	No

i=rank, m=number of tests, Q=false discovery rate of 0.10

© 2026 Pevnick JM et al. *JAMA Network Open*.

eTable 4. Effect of Intervention on Postdischarge Utilization Among Patients with Low Medication Adherence and Literacy. Two Multiple Imputation Models: One Unadjusted and One Adjusted for Potential Confounders.

Likelihood of Same-Hospital 30-day Utilization Among Patients with Low Medication Adherence and Literacy: Odds Ratio of Intervention Arm vs Usual Care Arm (n = 6238)		
Predictors	***Odds Ratio (95% Wald CIs)***	***p-value***
Unadjusted: Study arm, Medication Adherence and Literacy (low, non-low, or missing not thought to be at random)*	0.60 (0.40 - 0.88)	0.009
Adjusted: also accounting for Site, Age, Sex, Race, Ethnicity, Elixhauser, Native language, Insurance, Income, Number of prior admissions, and Number of chronic prescription medications**	0.61 (0.42 - 0.90)	0.010

* Multiple imputation for MedAL scores likely missing at random (patients enrolled at one site after MedAL stopped being used)
** Multiple Imputation also used for median income and number of medications

© 2026 Pevnick JM et al. *JAMA Network Open*.

eReference

Citation for PHARM-DC Study Protocol:

Pevnick JM, Keller MS, Kennelty KA, Nuckols TK, Ko EM, Amer K, Anderson L, Armbruster C, Conti N, Fanikos J, Guan J, Knight E, Leang DW, Llamas-Sandoval R, Matta L, Moriarty D, Murry LT, Muske AM, Nguyen AT, Phung E, Rosen O, Rosen SL, Salandanan A, Shane R, Schnipper JL. The Pharmacist Discharge Care (PHARM-DC) study: A multicenter RCT of pharmacist-directed transitional care to reduce post-hospitalization utilization. *Contemporary Clinical Trials*. 2021 Jul;106:106419. doi: 10.1016/j.cct.2021.106419. Epub 2021 Apr 28. PMID: 33932574; PMCID: PMC8276883.

© 2026 Pevnick JM et al. *JAMA Network Open*.
