# Supplementary material for: Pharmacist-Led Discharge Care to Reduce Postdischarge Health Care Utilization: A Randomized Clinical Trial
Source: JAMA Netw Open. 2026 Mar 17;9(3):e260719. doi: 10.1001/jamanetworkopen.2026.0719 (PMC13446754; doi:10.1001/jamanetworkopen.2026.0719)
Supplement: Supplementary file 3 [file jamanetwopen-e260719-s003.pdf]

Supplemental Online Content: Nonauthor Collaborators
*First name, last name, and suffix (if applicable) are required and will appear in PubMed.

| *Group Name(s): The PHARM-DC Group | | | | | | | |
|---|---|---|---|---|---|---|---|
| ***First Name and Middle Initial(s)** | ***Last Name** | ***Suffix (eg, Jr, III)** | Academic Degrees | Institution | Location (city, state/province, country) | Role or Contribution, eg, chair, principal investigator | Group (if more than 1 Group listed in the byline) and/or Subgroup (eg, Steering Committee) |
| Christine | Armbruster | | | Cedars-Sinai Medical Center | Los Angeles, California, USA | | The PHARM-DC Group |
| Nicole | Conti | | | Brigham and Women's Hospital | Boston, Massachusetts, USA | | The PHARM-DC Group |
| Michael | Corrado | | | Brigham and Women's Hospital | Boston, Massachusetts, USA | | The PHARM-DC Group |
| Ruby | Llamas-Sandoval | | | Cedars-Sinai Medical Center | Los Angeles, California, USA | | The PHARM-DC Group |
| Emily | Mai | | | Cedars-Sinai Medical Center | Los Angeles, California, USA | | The PHARM-DC Group |
| Sarah | Migeed | | | Brigham and Women's Hospital | Boston, Massachusetts, USA | | The PHARM-DC Group |
| Kelsey | Oneil | | | Brigham and Women's Hospital | Boston, Massachusetts, USA | | The PHARM-DC Group |
| Olga | Rosen | | | Cedars-Sinai Medical Center | Los Angeles, California, USA | | The PHARM-DC Group |
| Sonja | Rosen | | | Cedars-Sinai Medical Center | Los Angeles, California, USA | | The PHARM-DC Group |
| Madison | Smith | | | Brigham and Women's Hospital | Boston, Massachusetts, USA | | The PHARM-DC Group |
| William | Smith | | | Brigham and Women's Hospital | Boston, Massachusetts, USA | | The PHARM-DC Group |
| Lilly Kasha | Thao | | | Brigham and Women's Hospital | Boston, Massachusetts, USA | | The PHARM-DC Group |
| Jesse | Wisniewski | | | Cedars-Sinai Medical Center | Los Angeles, California, USA | | The PHARM-DC Group |
| Yi Tian | Xiao | | | Brigham and Women's Hospital | Boston, Massachusetts, USA | | The PHARM-DC Group |

Supplemental Online Content: Nonauthor Collaborators

*First name, last name, and suffix (if applicable) are required and will appear in PubMed.

| ***First Name and Middle Initial(s)** | ***Last Name** | ***Suffix (eg, Jr, III)** | Academic Degrees | Institution | Location (city, state/province, country) | Role or Contribution, eg, chair, principal investigator | Group (if more than 1 Group listed in the byline) and/or Subgroup (eg, Steering Committee) |
|---|---|---|---|---|---|---|---|
| Alexander | Chaitoff | | | University of Michigan | Ann Arbor, Michigan, USA | | The PHARM-DC Group |
