# Supplementary material for: Pharmacist-Led Discharge Care to Reduce Postdischarge Health Care Utilization: A Randomized Clinical Trial
Source: JAMA Netw Open. 2026 Mar 17;9(3):e260719. doi: 10.1001/jamanetworkopen.2026.0719 (PMC13446754; doi:10.1001/jamanetworkopen.2026.0719)
Supplement: Supplementary file 4 [file jamanetwopen-e260719-s004.pdf]

Data Sharing Statement

Pevnick. Pharmacist-Led Discharge Care to Reduce Postdischarge Health Care Utilization. *JAMA Netw Open*. Published March 12, 2026. doi:10.1001/jamanetworkopen.2026.0719

Data
Additional Information: Registered 8/26/2019 at https://clinicaltrials.gov/study/NCT04071951
Data available: No

Additional Information
Explanation for why data not available: Upon approval of the IRB, redacted data may be made available to interested investigators via communication with the study authors
